# Insights on Human Small Heat Shock Proteins and Their Alterations in Diseases

**DOI:** 10.3389/fmolb.2022.842149

**Published:** 2022-02-25

**Authors:** B. Tedesco, R. Cristofani, V. Ferrari, M. Cozzi, P. Rusmini, E. Casarotto, M. Chierichetti, F. Mina, M. Galbiati, M. Piccolella, V. Crippa, A. Poletti

**Affiliations:** ^1^ Dipartimento di Scienze Farmacologiche e Biomolecolari, Università degli Studi di Milano, Milan, Italy; ^2^ Unit of Medical Genetics and Neurogenetics, Fondazione IRCCS Istituto Neurologico Carlo Besta, Milan, Italy

**Keywords:** HSPBs, proteostasis, aggregation, neuropathy, muscle disease

## Abstract

The family of the human small Heat Shock Proteins (HSPBs) consists of ten members of chaperones (HSPB1-HSPB10), characterized by a low molecular weight and capable of dimerization and oligomerization forming large homo- or hetero-complexes. All HSPBs possess a highly conserved centrally located α-crystallin domain and poorly conserved N- and C-terminal domains. The main feature of HSPBs is to exert cytoprotective functions by preserving proteostasis, assuring the structural maintenance of the cytoskeleton and acting in response to cellular stresses and apoptosis. HSPBs take part in cell homeostasis by acting as holdases, which is the ability to interact with a substrate preventing its aggregation. In addition, HSPBs cooperate in substrates refolding driven by other chaperones or, alternatively, promote substrate routing to degradation. Notably, while some HSPBs are ubiquitously expressed, others show peculiar tissue-specific expression. Cardiac muscle, skeletal muscle and neurons show high expression levels for a wide variety of HSPBs. Indeed, most of the mutations identified in HSPBs are associated to cardiomyopathies, myopathies, and motor neuropathies. Instead, mutations in HSPB4 and HSPB5, which are also expressed in lens, have been associated with cataract. Mutations of HSPBs family members encompass base substitutions, insertions, and deletions, resulting in single amino acid substitutions or in the generation of truncated or elongated proteins. This review will provide an updated overview of disease-related mutations in HSPBs focusing on the structural and biochemical effects of mutations and their functional consequences.

## 1 Introduction

**FIGURE 1:**
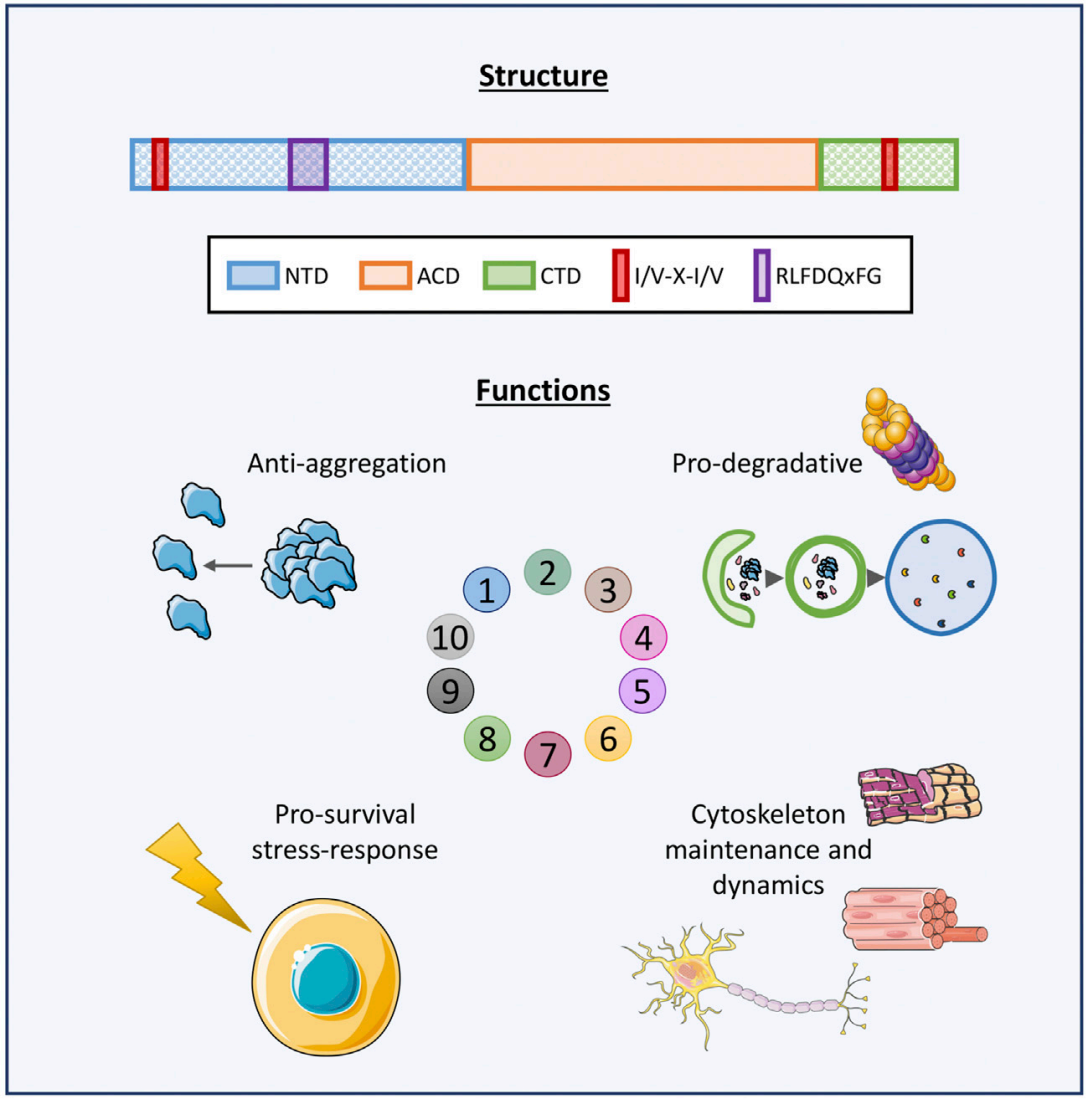
Graphical representation of HSPBs structure and main functions. Schematic representation of human HSPBs structure, from Cristofani et al. (2021). The orange box represents the conserved α-crystallin domain (ACD); the blue and green boxes represent the variable N-terminus and C-terminus domains (NTD, CTD, respectively); the red box indicates the I/V-X-I/V domains localizing at the NTD and/or CTD in several HSPBs; the purple box indicates the RLFDQxFG motif conserved in HSPBs. HSPBs functions include i) anti-aggregation activity against misfolded and aggregation-prone substrate and ii) cooperation with the degradative systems for substrate disposal, iii) protection against stressors and antiapoptotic activities, iv) cytoskeletal maintenance and dynamics. Servier Medical Art templates, which are licensed under a Creative Commons Attribution 3.0 Unported License were used in this figure; https://smart.servier.com.

Small Heat Shock Proteins (sHSPs or HSPBs) are a subset of chaperones characterized by low molecular weight (MW) (14–43 kDa), highly conserved and described in all kingdoms and also in some viruses (Caspers et al., 1995; Maaroufi and Tanguay, 2013). In mammals, HSPBs consist of ten members (HSPB1-HSPB10) (Table1) (Kappé et al., 2003; Kappé et al., 2010). HSPBs are structurally defined by the presence of an α-crystallin domain (ACD) in the central region of their sequence (Figure 1). The ACD is a highly conserved 80–90 amino acids (AA)-long domain consisting of 8 β-strands, which fold in a β-sandwich structure (van Montfort et al., 2001). Outside the ACD, HSPBs share a poor similarity of both the N-terminal and the C-terminal domains (NTD, CTD), except for the presence of a SRLFDQxFG motif in the NTD of the HSPBs B1, B4, B5, B6, B8, and a I/V-X-I/V motif in the CTD of HSPBs B1, B2, B4 and B5 (Pasta et al., 2003; Kriehuber et al., 2010; Poulain et al., 2010).

**TABLE 1:** Nomenclature and properties of HSPBs. The table lists HSPBs, their alternative names and properties. The molecular weight (MW), aminoacidic length (AA) and oligomerization propensity are reported. The presence of the SRLFDQxFG and the I/V-X-I/V motifs are reported. NTD and CTD refer to the N and C-terminal domains, respectively; N/A means not assessed.

HUGO name	Alternative names	MW (kDa)	AA	SRLFDQxFG motif	I/V-X-I/V	Assemblies
HSPB1	HSP27; HSP28	22,783	205	+	CTD	Oligomers
HSPB2	MKBP	20,233	182	−	CTD	Small oligomers
HSPB3	HSPL27	16,966	150	−	NTD	Small oligomers
HSPB4	αA-Crystallin; CRYAA	19,909	173	+	NTD/CTD	Oligomers
HSPB5	αB-Crystallin; CRYAB	20,159	175	+	NTD/CTD	Oligomers
HSPB6	HSP20; p20	17,136	160	+	NTD	Dimers
HSPB7	cvHSP	18,611	170	−	—	Dimers
HSPB8	HSP22; H11; E2IG1	21,604	196	+	—	Dimers
HSPB9	CT51	17,486	159	−	—	N/A
HSPB10	ODF1	28,366	250	−	−	N/A

HSPBs act as ATP-independent holdases, avoiding misfolded substrates aggregation (Leroux et al., 1997). Also, HSPBs indirectly interact with ATP-dependent chaperones with foldase activity, such as Heat Shock Protein 70 (HSP70/HSPA), for the refolding process (Jakob et al., 1993; Garrido et al., 2012). Another primary feature of HSPBs is their dynamic homo- or hetero-dimerization and oligomerization. Some HSPBs can form oligomers of 4 and up to 40 monomers, reaching MW of 900 kDa. All three ACD, NTD and CTD are fundamental for oligomerization and function. In particular, the ACD is responsible for dimer formation, while the NTD and the CTD promote the stabilization of oligomeric and dimeric structures. For instance, it has been observed that truncation of the NTD causes the formation of smaller HSPBs oligomers, while the absence of the I/V-X-I/V motif in the CTD of HSPBs B3, B6, B8 is thought to explain their low oligomerization propensity (Boelens, 2020). In response to environmental stimuli, a dynamic change in subunit composition of oligomers can occur, changing the affinity for substrates binding, and therefore the functional activity of oligomers (van Montfort et al., 2001; Basha et al., 2012; Mymrikov et al., 2020). HSPBs exert several activities in cells (Figure 1). First, HSPBs, acting as holdase chaperones, prevent protein aggregation and help in refolding. Second, HSPBs promote substrates routing to the degradative pathways, autophagy and the Ubiquitin Proteasome System (UPS) (Parcellier et al., 2006; Carra et al., 2008a; Zhang et al., 2010). Third, HSPBs interact with the cytoskeleton components regulating their assembly and assuring structural integrity (Wettstein et al., 2012). Fourth, HSPBs are key players in the response to cellular stress and apoptosis, by regulating enzymes involved in oxidative stress response, participating in redox metabolism and blocking the activity of pro-apoptotic factors (Arrigo, 2007; Acunzo et al., 2012).

**FIGURE 2:**
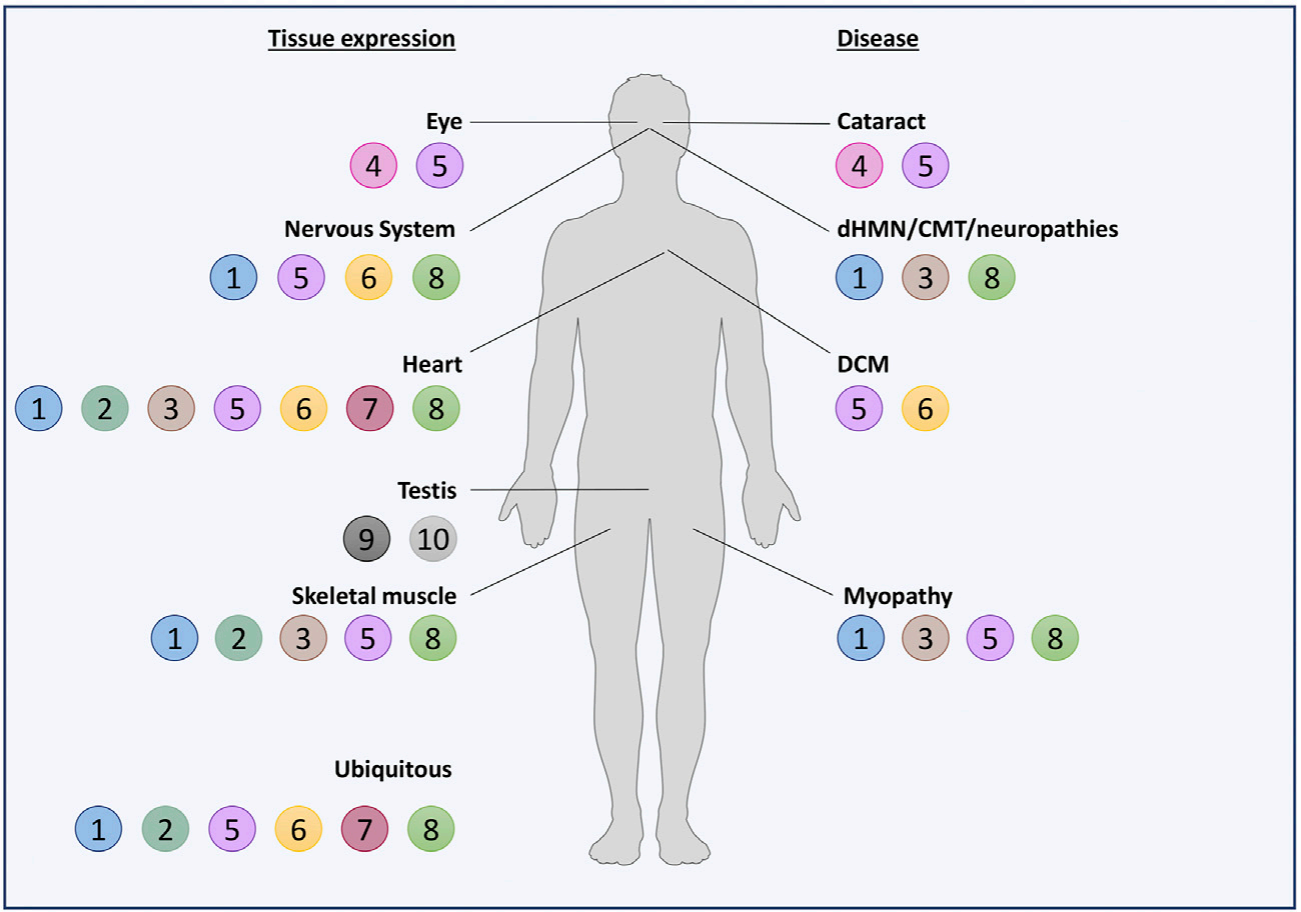
Schematic representation of HSPBs tissue expression and related diseases. HSPBs are represented as beads and numbered based on HUGO nomenclature. On the left, HSPBs tissue expression is reported: most of HSPBs are expressed ubiquitously, but predominantly in certain tissues; other HSPBs show a tissue-specific expression. On the right, diseases in which HSPBs mutations have been identified are reported. Servier Medical Art templates, which are licensed under a Creative Commons Attribution 3.0 Unported License, were used in this figure; https://smart.servier.com.

**FIGURE 3:**
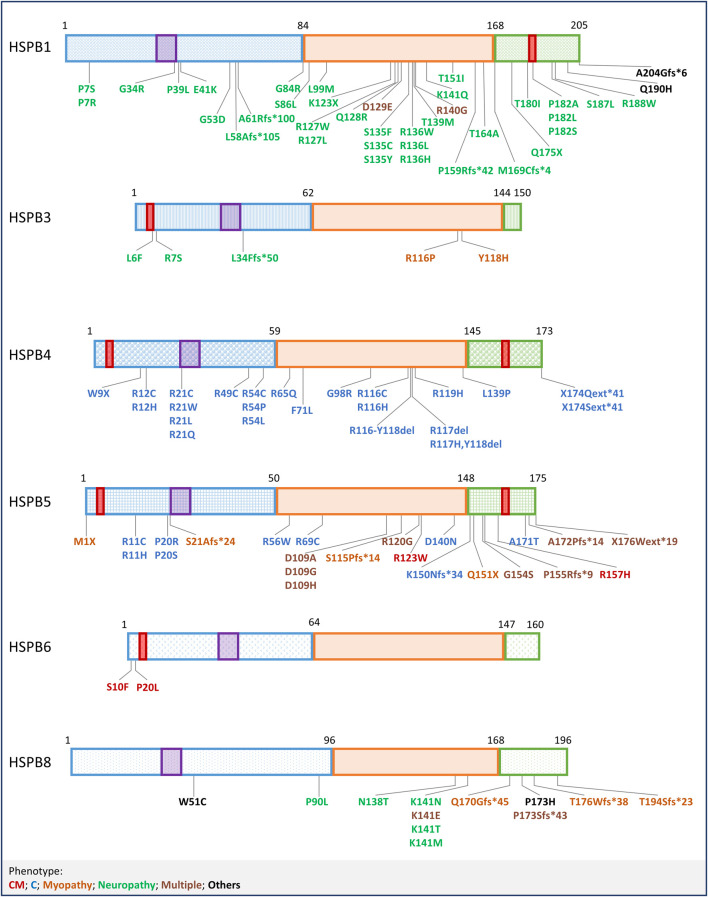
Mutations in HSPBs. Graphical representation of HSPBs structure and mutations, based on Figure 1. Mutations are labelled with colors corresponding to the phenotypes reported at the bottom. CM = Cardiomyopathy, C=Cataract; Multiple (phenotypes) refers to mutations that have been linked to more than one phenotype, in combination or not; Others refer to mutations not included in the list.

Noteworthily, half of the HSPBs (B1, B2, B5, B6, B7, and B8) are ubiquitously expressed, while the others show a tissue-specific expression (Figure 2). However, also the ubiquitously expressed HSPBs show higher levels in certain cell types, such as neurons, skeletal and cardiac muscle cells, suggesting a need of these cells for selective HSPBs activities. In fact, mutations in HSPBs affect these cells, causing neuropathies, myopathies, and/or cardiomyopathies (Figure 3; Supplementary Material).

Here we will discuss the general and the specific properties of each HSPB, underlining how they selectively contribute to the typical response to cell stresses and how the reported mutations impact on their structure, stability, and function in disease.

## 2 HSPB1

HSPB1 is a 23 kDa protein constitutively and widely expressed in all human tissues. HSPB1 has been first described upregulated in HeLa cells upon heat shock (Arrigo and Welch, 1987). HSPB1 is mainly found in polydisperse oligomers of 400–600 kDa and its phosphorylation, dimerization, and oligomerization have been extensively studied (Arrigo, 2017). HSPB1 dimerization can be affected by the intracellular redox conditions, which modulate the formation of a disulphide bond between cysteine residues (C137) at the dimer interface in the β7 strand of the ACD (Zavialov et al., 1998; Diaz-Latoud et al., 2005; Mymrikov et al., 2010; Baranova et al., 2011; Chalova et al., 2014a; Rajagopal et al., 2015; Alderson et al., 2019). HSPB1 exerts several functions under physiological or stressed conditions: it indirectly cooperates in refolding or degradation of substrates through the UPS or autophagy (Zhang et al., 2010; Minoia et al., 2014; Haidar et al., 2019; Gonçalves et al., 2021); it interacts with cytoskeletal elements, modulating their correct assembly and preventing their damage (Miron et al., 1991; Perng et al., 1999); it plays an anti-apoptotic activity at multiple levels, by interfering with the pro-apoptotic proteins Bax and Bid and by sequestering the cytochrome c released from mitochondria, hence inhibiting the caspase cascade (Bruey et al., 2000; Pandey et al., 2000; Paul et al., 2010). All HSPB1 activities reflect its role in protection against several stressors such as thermal, oxidative, and proteotoxic stress. In the nervous system, HSPB1 protects cells from misfolded protein toxicity exerted by disease-associated mutant proteins, such as Huntingtin (HTT) carrying an elongated polyglutamine (polyQ) tract, the mutated Superoxide Dismutase 1 (SOD1) and α-synuclein (SNCA), which are able to cause neurodegeneration (Wyttenbach et al., 2002; Zourlidou et al., 2004; An et al., 2009). HSPB1 acts also in a non-cell autonomous fashion in neuroprotection. Indeed, HSPB1 upregulation has been described in astrocytes derived from transgenic (TG) mice carrying a mutated form of SOD1, while its overexpression promotes motoneurons (MNs) survival (Heilman et al., 2017). In skeletal and cardiac muscle, HSPB1 protects the cytoskeleton from structural damage (Lavoie et al., 1995; Bryantsev et al., 2002; Collier et al., 2019).

It is thus extremely relevant to underline that more than 30 mutations related to neuromuscular diseases have been identified in HSPB1, representing the most frequent cause of hereditary neuropathies, like distal Hereditary Motor Neuropathy type II (dHMNII) and Charcot-Marie-Tooth type 2 (CMT2) diseases. These disorders are characterized by adult onset and slow progression rate, but patients carrying HSPB1 mutated gene may show additional and more severe symptoms beside motor neuropathy, such as sensory involvement, cerebellar ataxia, spasticity and myopathic features. Some HSPB1 mutations even associate with a rapidly progressive phenotype that resembles Amyotrophic Lateral Sclerosis (ALS) (Capponi et al., 2016). HSPB1 mutations are mainly inherited dominantly and occur along the whole HSPB1 sequence. Despite the well evaluated clinical phenotypes, the biochemical and functional characterization for most of these HSPB1 mutations is still missing, and the molecular mechanisms at the basis of HSPB1-related neuropathies are not straightforward. Nevertheless, efforts have been made to decipher the dysfunctions at the basis of this multitude of HSPB1 mutations. Since the major aspect regulating HSPB1 activity relies on its self-association and dissociation, dimerization and oligomerization dynamics have been investigated as one of the primary features possibly altered in the mutant protein. *In vitro*, biochemical studies on HSPB1 mutations falling in different regions of the protein sequence revealed a common tendency to form larger oligomers, often accompanied by a decreased chaperone activity in respect to the HSPB1 wild-type (WT); *in vitro* analyses also reported a destabilization of the quaternary structure of mutated HSPB1 (Nefedova et al., 2013a; Nefedova et al., 2013b; Chalova et al., 2014b; Muranova et al., 2015; Alderson et al., 2021). Clustering HSPB1 mutations through a domain-dysfunction linkage is not unequivocal, due to contrasting observations resulted from different techniques and assays used to study mutations localized in the same HSPB1 region (Almeida-Souza et al., 2010; Ylikallio et al., 2015; Echaniz-Laguna et al., 2017a). Nevertheless, it is well assumed that mutations falling in the ACD might affect dimerization. Alterations in oligomerization and aberrant interaction with other proteins may explain the presence of aggregates of HSPB1 mutants observed in cells expressing different HSPB1 mutants (Evgrafov et al., 2004; Ackerley et al., 2006; James et al., 2008; Echaniz-Laguna et al., 2017a). Aggregation of HSPB1 mutants has a double consequence on cell homeostasis: from one side, HSPB1 mutants can sequester the HSPB1 WT, acting in a dominant negative manner and resulting in a loss of protective activity of the HSPB1 protein; from the other side, protein aggregates *per se* alter several cellular functions, e.g., proteostatic mechanisms, cytoskeletal architecture maintenance and axonal transport. Despite HSPB1 self-aggregation, which has not been observed for all HSPB1 mutations, the above-mentioned functions have been found altered in several cell models expressing HSPB1 mutants. For instance, while the HSPB1 WT promotes autophagy, HSPB1 mutations associate with a decreased autophagic flux (Amornvit et al., 2017; Haidar et al., 2019). Regarding cytoskeletal architecture maintenance, HSPB1 mutants were described to alter both intermediate filaments and microtubule network. The effects of HSPB1 mutants on neurofilaments (NF) assembly and organization are variable, from a slight increase in NF phosphorylation to a severe co-aggregation with the HSPB1 mutants (Zhai et al., 2007; Holmgren et al., 2013; Echaniz-Laguna et al., 2017a). Loss of the typical filamentous network of NF was observed in cells expressing the HSPB1 S135F mutant, which co-aggregated with NF at the perinuclear region (Evgrafov et al., 2004). Similarly, a disrupted and co-aggregating NF network was observed in primary neurons expressing the HSPB1 P182L mutation (Ackerley et al., 2006). Alterations of microtubule network resulted in a disrupted or defective axonal transport of organelles, another hallmark of axonal neuropathies (Ackerley et al., 2006; Kalmar et al., 2017). Given the multiple roles of HSPB1 in cell homeostasis, it is not surprising that increased cytotoxicity and decreased resistance against stressors were observed in cells expressing HSPB1 mutants (Evgrafov et al., 2004; Ylikallio et al., 2015; Kalmar et al., 2017). Truncation and frameshift mutations in HSPB1 have been identified. Experimental findings suggest that these mutated HSPB1 variants are still expressed but affected by protein instability. Analyses on cells overexpressing the HSPB1 frameshift mutants L58Afs*105 and A61Rfs*100 revealed that these proteins are less stable than the HSPB1 WT and rapidly degraded through the proteasome (DiVincenzo et al., 2014; Echaniz-Laguna et al., 2017a). Instead, the HSPB1 M169Cfs*4 mutation, which results in the production of a protein lacking almost the entire CTD, is still expressed in patient fibroblasts. While the mutant HSPB1 M169Cfs*4 distribution seemed unaffected, functional analyses revealed that patient-derived fibroblasts are less tolerant to stress stimuli, and subjected to an increase in misfolded protein load in respect to control cells (Ylikallio et al., 2015). Similar findings have been reported for the HSPB1 A204Gfs*6, a rare variant described in ALS patients. HSPB1 A204Gfs*6 is less stable than the HSPB1 WT but it still dimerizes sequestering the HSPB1 WT. This causes an impairment in HSPB1-mediated clearance of misfolded substrates (Capponi et al., 2016). Overall, these observations suggest that, even if HSPB1 protein stability might be affected by truncating and frameshift mutations, their expression might result in a dominant negative action on the HSPB1 WT, which correlates with a defective cytoprotective activity. Interestingly, HSPB1 deficiency in mice is not linked to a neurological phenotype: in a HSPB1 Knock-out (KO) mouse model neither association to a neuromuscular phenotype nor developmental defects were observed (Huang et al., 2007); while in a second HSPB1 KO mouse, muscular impairments were observed in absence of neurological signs. Overall, these HSPB1 KO mice showed neither developmental defects nor alteration during aging, but a slight decrease in body weight. Ultrastructural studies revealed defects in the structural organization of muscle fibres, consisting in increased intermyofibrillar spaces and alterations in myofibrillar arrangement (Kammoun et al., 2016). Skeletal muscle targeting by HSPB1 is not surprising since HSPB1 mutations (e.g., D129E and R140G) have been described in patients showing myopathy features (Lewis-Smith et al., 2016; Bugiardini et al., 2017). Some HSPB1 mutations have been extensively studied in animal models. Two TG mouse models expressing the HSPB1 S135F mutant have been developed: these mice were vital at birth and manifested motor defects. One of the two mouse models expressed the HSPB1 S135F mutation selectively in neuronal tissue and at 6 months began to manifest progressive motor impairments and signs of sensory defects. Electrophysiological changes, but not demyelination, were observed, revealing an axonal neuropathy. Isolated dorsal root ganglion neurons displayed an impaired mitochondrial axonal transport and reduced acetylated tubulin, suggesting a destabilization of the microtubule network. Noteworthily, HDAC6 inhibitors, by counteracting the deacetylation of tubulin and therefore stabilizing the microtubule network, enhanced motor performance of these mice (d’Ydewalle et al., 2011). The other TG mouse model expressing the human HSPB1 S135F showed reduced motor function at 5 months, correlating with alteration in nerve conduction, a reduction in myelinated axons and fatty acid replacement in muscle. Analyses on sciatic nerves highlighted a reduction in tubulin acetylation and increased levels of phosphorylated NFs (Lee et al., 2015). The TG fly (*Drosophila melanogaster*) expressing the human HSPB1 S135F mutant in neurons or specifically in MNs did not show developmental defects or decreased life span, but a deeper analysis revealed motor defects, which were reverted by HDAC6 downregulation (Kang et al., 2020). Another TG mouse model expressing human HSPB1 R136W in neurons did not show an evident motor phenotype, but electrophysiological and histological analyses revealed signs of neuropathy (Srivastava et al., 2012). TG mice expressing the HSPB1 P182L mutation in the nervous system developed normally until 6 months of age, when they began manifesting motor impairments, axon loss and denervation (d’Ydewalle et al., 2011). Conversely, other TG mouse models did not recapitulate the neuropathy phenotype, an outcome likely related to a low transgene expression. For instance, mouse models expressing HSPB1 R127W and P182L mutants at levels comparable to the physiological ones did not show motor impairment nor signs of axonal damage (Bouhy et al., 2016).

## 3 HSPB2

HSPB2 has a MW of 20 kDa. HSPB2 gene locates near the HSPB5 gene, but in the opposite direction in the 5′ flanking region. Given this unique feature, it has been speculated that HSPB2 gene derives from an ancestral gene duplication (Iwaki et al., 1997). HSPB2 was identified by two independent groups that named it HSPB2 or Myotonic dystrophy protein Kinase Binding Protein (MKBP) (Iwaki et al., 1997; Suzuki et al., 1998). The MKBP name was given since HSPB2 was described as an interactor and activator of DMPK, a kinase that physiologically acts in muscle structure maintenance and can be deregulated in myotonic dystrophy. Indeed, HSPB2 has been observed upregulated in myotonic dystrophy patients (Suzuki et al., 1998). Both groups observed that, even if ubiquitously expressed, HSPB2 is particularly elevated in skeletal and cardiac muscle (Iwaki et al., 1997; Suzuki et al., 1998). For instance, HSPB2 increases its expression in C2C12 myoblasts during differentiation into myotubes, likely under the control of the myogenic factor MyoD (Sugiyama et al., 2000; Nakagawa et al., 2001). HSPB2 is not heat-inducible, but heat shock itself reduces HSPB2 solubility (Iwaki et al., 1997; Suzuki et al., 1998). As seen for the other HSPBs, HSPB2 overexpression increases cell survival after heat stress (Nakagawa et al., 2001). Other stressors or pathological conditions may induce HSPB2 upregulation and/or redistribution as a part of the intracellular protective mechanism against stress. Proteasome impairment induces HSPB2 delocalization to actin fibres and, similarly, ischemia-mediated stress and oxidative stress induce HSPB2 translocation to Z-lines and intercalated discs in cardiomyocytes (Shama et al., 1999; Yoshida et al., 1999; Verschuure et al., 2002). In skeletal muscle of symptomatic mouse models of Spinal and Bulbar Muscular Atrophy (SBMA), HSPB2 is upregulated (Rusmini et al., 2015). In the nervous system, HSPB2 has been detected in extracellular plaques and cerebral amyloid angiopathy in Alzheimer’s disease (AD) patients (Wilhelmus et al., 2006c). In cells, HSPB2 localization was observed in nuclei, where it tends to form droplets in a concentration-dependent manner through a liquid-liquid phase separation (LLPS) process (Morelli et al., 2017b). The CTD of HSPB2 drives its LLPS since its deletion prevents the formation of HSPB2 nuclear foci. The enhancement of this process, which might be a consequence of HSPB2 overexpression but also of the loss of regulatory activity of HSPB2-partner HSPB3, results in the deregulation of chromatin distribution and in the loss of nuclear integrity (Morelli et al., 2017b). HSPB2 forms a 3:1 complex with HSPB3, which represents the building block for further self-interactions (den Engelsman et al., 2009; Clark et al., 2018). HSPB3, by interacting with HSPB2, regulates its activities and interactions with other partners (Morelli et al., 2017a). Also, HSPB2 has been described to interact with HSPB6 (den Engelsman et al., 2009). Results on HSPB2 chaperone activity are controversial: in some studies HSPB2/HSPB3 complex was reported to exert no or mild anti-aggregation activity against substrates prone to fibrillization such as Amyloid β (Aβ) or mutated SNCA both *in vitro* and in cells (Wilhelmus et al., 2006b; Bruinsma et al., 2011), while other studies on HSPB2 alone reported an effective chaperone activity (Prabhu et al., 2012; Minoia et al., 2014). Although this discrepancy might be related to the differences in the assays utilized, another explanation is that HSPB2 activity might be dependent on its oligomerization state with HSPB3 (Clark et al., 2018).

HSPB2 mutations have not been identified so far. Interestingly, by developing an HSPB5 KO mouse, given the proximity of the two genes, double KO (DKO) mice for HSPB5 and HSPB2 have been generated. These mice did not show developmental defects or alterations related to HSPB5 specific functions in eyes and HSPB2/HSPB5 activities in heart. Instead, muscle degeneration, loss of body weight, and severe postural defects were observed in these DKO aging mice (Brady et al., 2001). Subsequent studies revealed that, even if cardiac functions and development were unaffected, HSPB2/HSPB5 DKO mouse hearts showed increased cell death and defective recovery upon Ischemia/Reperfusion (I/R) tests, likely related to the loss of protective function of the two HSPBs towards cytoskeletal contractile elements (Morrison et al., 2004; Golenhofen et al., 2006). Unfortunately, in these initial studies, it was not possible to discriminate if the phenotype observed was related to one or the other HSPBs, since both HSPBs are relevant in the maintenance of skeletal muscle. However, subsequent studies were able to dissect the different roles of the two neighbour genes in heart. Indeed, by comparing DKO mice with DKO animals that were genetically modified to re-express HSPB5, alterations in cardiac energetics have been observed and related to HSPB2 absence (Pinz et al., 2008). These alterations consisted in a faster rate of ATP loss during ischemia, a decreased energy recovery during reperfusion and an inefficient coupling of ATP hydrolysis-derived free energy and work (Pinz et al., 2008). On the other hand, HSPB2 overexpression in cardiac tissue in mice associated with a reduction in the size of infarcted tissues after I/R (Grose et al., 2015). The involvement of HSPB2 in metabolism and mitochondrial energetics was additionally proven in a cardiac-specific HSPB2 KO mouse (Ishiwata et al., 2012). In support to the HSPB2 role in energetics, HSPB2 mitochondrial localization was reported to be enhanced after heat shock and cardiac-specific HSPB2 KO mouse model revealed an altered mitochondrial activity in response to stress (Nakagawa et al., 2001; Ishiwata et al., 2012).

## 4 HSPB3

HSPB3 gene was initially identified from a human heart cDNA library (Lam et al., 1996). The correct sequence of HSPB3 was subsequently defined by Boelens et al. (1998), who described the presence of the HSPB3 transcript in smooth muscle, heart and several foetal tissues. HSPB3, with a MW of 17 kDa, is the smallest HSPB. *In vitro*, HSPB3 is found either in trimers or in tetramers (Asthana et al., 2012). In cells, HSPB3 oligomerizes with HSPB2 forming a 1:3 stoichiometric complex (Sugiyama et al., 2000; den Engelsman et al., 2009). Notably, HSPB3 chaperone-like activity is moderate *in vitro*, and most of its functions concern its regulatory activity on HSPB2. For instance, HSPB3 oligomerization with HSPB2 negatively regulates HSPB2 interaction with BAG3 (Morelli et al., 2017a). HSPB3 also inhibits HSPB2 recruitment into nuclear foci (Morelli et al., 2017b). HSPB3 is mainly expressed in cardiac and skeletal muscle, but also in smooth muscle and nervous system. In C2C12 myoblasts, like HSPB2, HSPB3 is expressed with differentiation (Sugiyama et al., 2000). Indeed, HSPB3 gene presents a MyoD-responsive region and HSPB3 overexpression induces myoblasts differentiation (Tiago et al., 2021). Instead, HSPB3 expression in the nervous system is moderate and varies during development. HSPB3 is found in glial cells, in MNs and in sensory neurons (La Padula et al., 2016). HSPB3 localizes in the cytoplasm and associates to cytoskeletal components, e.g., actin bundles and NFs, but also mitochondrial membrane and nuclear envelope (La Padula et al., 2016; Tiago et al., 2021). HSPB3 is upregulated upon proteotoxic stress but not upon thermal stress. For instance, as HSPB2, HSPB3 is upregulated in skeletal muscle of a symptomatic mouse model of SBMA, supporting the specific role of these two HSPBs in muscle maintenance upon proteotoxicity (Rusmini et al., 2015).

HSPB3 mutations have been linked to neuropathies and myopathies. The first reported mutation consists in the substitution R7S identified in dHMNII (Kolb et al., 2010; Laššuthová et al., 2016). The HSPB3 R7 residue is well conserved among the HSPB3 orthologues but not among HSPBs, suggesting a functional role of the residue in HSPB3 activity (Kolb et al., 2010; Benndorf et al., 2014). However, the HSPB3 R7S mutation does not alter the HSPB2-HSPB3 hetero-dimerization, it has a mild impact on oligomerization and functional defects have not been described, yet (La Padula et al., 2016; Clark et al., 2018). Mutations located in the ACD of HSPB3 are the substitutions R116P and Y118H (Morelli et al., 2017b; Nam et al., 2018). The HSPB3 R116P mutation has been reported in a patient affected by myopathy with axonal neuropathy and in her father affected by mild neuromuscular symptoms (Morelli et al., 2017b). Analyses of the HSPB3 R116P patient muscle biopsies revealed a severe myofibrillar disorganization affecting the Z-disc, abnormal sarcoplasmic reticulum morphology, pluri-segmented nuclei, and intermyofibrillar glycogen accumulation (Morelli et al., 2017b). The HSPB3 R116 residue is conserved among HSPB3 orthologues and it has been postulated that it is topologically equivalent to the R140 residue in HSPB1 (Morelli et al., 2017b; Clark et al., 2018). In cells, the HSPB3 R116P mutant, by losing the ability to bind HSPB2, is not able to regulate the typical HSPB2 nucleus-cytoplasm distribution and thus, associates to the abnormal HSPB2 nuclear LLPS (Morelli et al., 2017b). Another HSPB3 R116P pathogenic mechanism relies on its intranuclear aggregation, which results in HSPB3 WT sequestration and deregulation of transcription processes (Morelli et al., 2017b; Tiago et al., 2021). Instead, the HSPB3 Y118H mutation has been described in one CMT patient with a family history of disease. Analysis of HSPB3 orthologues indicates that the Y118 residue has been conserved during evolution (Nam et al., 2018). Similar to R116P, also HSPB3 Y118H mutation is located at the dimerization surface of the ACD and might determine the loss of interaction with the HSPB2 partner (Morelli et al., 2017b; Nam et al., 2018). However, biochemical and functional studies on HSPB3 Y118H homo- and hetero-oligomerization and activity are still missing. The HSPB3 A33AfsX50 (L34Ffs*50) frameshift mutation has been identified in a 70-years-old patient affected by myopathy (Morelli et al., 2017b). Studies in cells revealed that this frameshift mutant, which causes a truncation of the HSPB3 protein, is unstable and rapidly subjected to proteasomal degradation. This results in the loss of regulatory activity on HSPB2, which eventually causes aberrant nuclear foci formation (Morelli et al., 2017b). Finally, from the analysis of a small cohort of CMT/dHMN patients, a novel variant of unknown significance, the HSPB3 L6F, has been recently identified in a patient affected by dHMNII (Yalcintepe et al., 2021). To our knowledge, animal models of HSPB3 mutants have not been developed.

## 5 HSPB4 and HSPB5

HSPB4 and HSPB5 are highly expressed in lenses. Lens fibres are post-mitotic cells characterized by the absence of most of the light-scattering intracellular organelles, such as nuclei and mitochondria, to guarantee the lens transparency (Bassnett, 2009). The most abundant proteins in lens are α-, β-, γ- crystallins and form the liquid-like structures of lenses. To maintain lens transparency, the α-crystallins HSPB4 and HSPB5, which represent one-third of total soluble proteins in lens, exert an anti-aggregating activity against proteins that could be damaged during aging (Horwitz, 1992; Andley, 2007). In lenses, HSPB4 and HSPB5 interact with cytoskeletal components: they prevent incorrect interactions of intermediate filaments, avoiding gel formation; they protect actin fibres against stress and participate in actin dynamics in lamellipodia in lens epithelial cells; they stabilize the microtubule network (Del Vecchio et al., 1984; Maddala and Rao, 2005; Xi et al., 2006). Also, HSPB4 and HSPB5 exert a pro-survival activity upon thermal and oxidative stresses or UV-light damage (Andley et al., 1998; Christopher et al., 2014). Indeed, HSPB4 and HSPB5 both abrogate UVA-induced apoptosis by activating the AKT pathway and suppressing the RAF-MEK-ERK pathway, respectively (Liu et al., 2004). Moreover, both HSPB4 and HSPB5 inhibit the pro-apoptotic proteins Bax, Bcl-Xs and caspase-3 (Andley, 2007). Interestingly, HSPB4 expression is confined in lenses and the protein is synthesized later in respect to HSPB5 during cell differentiation (Robinson and Overbeek, 1996). Instead, HSPB5 is also expressed in skeletal and cardiac muscle cells. In cardiac muscle, HSPB5 assures the correct folding of titin, a structural protein involved in muscle fibres integrity, and HSPB5 overexpression associates with less cardiac damage and better contractile functions (Golenhofen et al., 1999; Bullard et al., 2004). In skeletal muscle, HSPB5 expression increases with aging (Doran et al., 2007). HSPB5 is also expressed in the central nervous system and its expression increases during life. HSPB5 exerts an anti-aggregation activity toward different misfolded substrates, such as mutated SNCA, SOD1, and Aβ (Waudby et al., 2010; Shammas et al., 2011; Yerbury et al., 2013; Cox and Ecroyd, 2017). HSPB5 neuroprotective function seems to be mediated by glial-related mechanisms (Bajramović et al., 2000; Ousman et al., 2007; Hagemann et al., 2009; Oliveira et al., 2016; Gorter et al., 2019; Hampton et al., 2020).

Mutations in HSPB4 and HSPB5 have been related to cataracts, myopathies, and cardiomyopathies, based on their tissue expression profile. Most of the mutations are autosomal dominant. As mentioned above, HSPB4 mutations strictly impact eye functions, ranging from more severe phenotypes affecting subjects at birth to less severe and late onset conditions. A rare example of HSPB4 mutation characterized by recessive inheritance is the nonsense mutation HSPB4 W9X, causing congenital cataracts. Indeed, the phenotype manifests only in homozygosis, suggesting that the WT allele is sufficient to compensate the loss of the mutated allele (Pras et al., 2000). HSPB4-deficient models partially resemble this phenotype. Indeed, HSPB4 KO mice do not manifest an obvious phenotype at birth, but, with aging, progressive lens opacity and the presence of inclusion bodies in lenses are observed. Interestingly, the full depletion of the HSPB4 protein associates with poor solubility of the HSPB5 partner and of other “crystallin” proteins, a feature not observed in the heterozygous mouse (Brady et al., 1997). Several HSPB4 mutations result in substitutions of arginine residues throughout the protein sequence. Similarly to HSPB1, most of HSPB4 missense mutations affect oligomerization, solubility, and aggregation properties (Mackay et al., 2003; Santhiya et al., 2006; Andley et al., 2008; Sun et al., 2011; Laurie et al., 2013; Yang et al., 2013; Khoshaman et al., 2017; Song et al., 2018). Of interest, a peculiar behaviour has been related to arginine-to-cysteine substitutions: indeed, *in vitro* biochemical studies demonstrated that HSPB4 R12C and R54C assemble into high MW oligomers or aggregates through the formation of disulphide bonds between monomers (Khoshaman et al., 2015; Khoshaman et al., 2017). Other HSPB4 mutations cluster at residues 116–119 (Clark et al., 2011). Even in this case, most of these HSPB4 mutants associate with an impairment in oligomerization and aggregation propensity (Litt et al., 1998; Li et al., 2010; Pang et al., 2010; Zhang et al., 2011). Interestingly, not only missense substitutions but also in-frame deletions of these hot-spot AA determine HSPB4 aggregation (Li et al., 2017). As a consequence of HSPB4 aggregation, activation of stress signalling pathways is observed. For instance, activation of the unfolded protein response (UPR) signalling in response to misfolded protein overload, autophagy impairments, and endoplasmic reticulum (ER) stress, were observed and correlated to an increased occurrence of apoptotic events (Andley et al., 2002; Li et al., 2017). Mouse models carrying HSPB4 mutations recapitulate well the human phenotype. For instance, HSPB4 decreased solubility and aggregation were observed in a Knock-in (KI) mouse model expressing the HSPB4 R49C mutation (Xi et al., 2008). Noteworthily, both the heterozygous and the homozygous mouse models showed lens opacity, more severe for the latter. Homozygous mice showed also severe microphthalmia, with halved lens weight. The pathogenic phenotype of the homozygous mice, unlike the heterozygous ones, was present at birth and might result from early defects during lens development at embryonic stages (Andley and Goldman, 2016). Indeed, analyses on homozygous mouse lenses at embryonic stages and at birth showed severe morphological alteration, characterized by lens fibre cells disorganization, defective cell-cell interactions, and vacuoles formation (Xi et al., 2008; Andley and Goldman, 2016). Further investigations in mice carrying the HSPB4 R49C mutation revealed an impairment of the autophagic pathway, as shown by sequestosome1/p62 (SQSTM1/p62) accumulation and defective autophagosomes, and UPR pathway activation (Watson and Andley, 2010; Andley and Goldman, 2016). The HSPB4 R54H and R54C substitutions have been also detected in mice strains showing recessive congenital cataract (Chang et al., 1996; Chang et al., 1999; Xia et al., 2006). Another mutation, the HSPB4 Y118D substitution, has been obtained in two mouse models (Xia et al., 2006; Jia et al., 2021). Both HSPB4 Y118D mouse models recapitulate the phenotype and the biochemical and functional observations reported, which are the increased protein insolubility and UPR pathway activation in response to ER stress (Xia et al., 2006; Li et al., 2017; Jia et al., 2021). Mutations causing substantial modification of the CTD include the HSPB4 Q147Rfs*48 frameshift mutation or point mutations at the stop codon (Javadiyan et al., 2017; Marakhonov et al., 2020). The latter are the HSPB4 *174Qext*41 and *174Sext*41 *de novo* mutations, reported in patients suffering from congenital aphakia, a developmental condition characterized by lens absence, microphthalmia, microcornea, and iris hypoplasia/aniridia (Marakhonov et al., 2020). These two mutations, falling in the stop codon of the HSPB4 gene, cause the abrogation of the translation termination and the production of an elongated protein. The pathogenic mechanisms at the basis of these mutations have not been investigated, but it has been suggested that the presence of the aberrant C-terminal extensions impact on HSPB4 solubility and chaperone function, abolishing its cytoprotective activities (Marakhonov et al., 2020). Given the role of HSPB4 in lenses, HSPB4 polymorphisms have been identified at the promoter or untranslated regions of the HSPB4 gene in association with milder pathogenic conditions such as age-related cataract and macular degeneration, but the pathogenicity remains uncertain (Ma et al., 2016; Xu et al., 2019; Hongtao Yu et al., 2021).

Similarly to HSPB4, several mutations of HSPB5 have been identified in congenital cataracts and most of these mutations associate with increased aggregation propensity (Li et al., 2008; Raju and Abraham, 2013; Ghahramani et al., 2020). As observed for HSPB4, several of these HSPB5 mutations consist in arginine substitutions, which affect HSPB5 homo- and hetero- oligomerization and chaperone activity (Raju and Abraham, 2013; Ghahramani et al., 2020; Muranova et al., 2020). Aggregation is also a feature of HSPB5 mutants linked to myopathies, cardiomyopathies or more severe multisystem disorders (Vicart et al., 1998; Inagaki et al., 2006; Reilich et al., 2010; Brodehl et al., 2017; Fichna et al., 2017). Aggregation has been also observed in HSPB5 mutations at residues R120 and D109, which are considered to mediate HSPB dimerization, by forming a salt bridge (Simon et al., 2007b; Michiel et al., 2009; Clark et al., 2011). HSPB5 R120G substitution was the first HSPB mutation to be linked to a neuromuscular disease and thus it has been deeply characterized (Vicart et al., 1998). Indeed, HSPB5 R120G cytoplasmic aggregates have been detected in several cell models, localizing at the perinuclear region in aggresomal structures positive for amyloid oligomers (Vicart et al., 1998; Chávez Zobel et al., 2003; Sanbe et al., 2004; Inagaki et al., 2006; Nivon et al., 2016; Brodehl et al., 2017). TG mice overexpressing the HSPB5 R120G mutation specifically in heart showed a 100% mortality at adult age due to cardiac hypertrophy and dysfunction. In agreement with cell studies, the HSPB5 R120G has been found in aggregates together with desmin, a muscle-specific intermediate filament, in mouse hearts (Wang et al., 2001). Beside HSPB5 R120G effect on structural proteins, other functional alterations were observed, including aberrant mitochondrial activity, ER stress, calcium homeostasis dysregulation, and apoptosis, likely related to HSPB5 aggregation-mediated proteotoxicity (Maloyan et al., 2005; Jiao et al., 2014; Alam et al., 2020). Indeed, proteostasis impairment characterized both TG mice and cells overexpressing the HSPB5 R120G mutant, as supported by an increase in ubiquitinated proteins and defective delivery of substrates to the UPS (Chen et al., 2005). Interestingly, an increase of the autophagic flux was also described, which might represent a compensatory mechanism to counteract the misfolded protein overload (Tannous et al., 2008). Indeed, autophagic inhibition both in cells and *in vivo* resulted in a worsening of HSPB5 R120G phenotype, while its potentiation favoured HSPB5 aggregates removal (Tannous et al., 2008; Pattison et al., 2011). Conversely, HSPB5 deficiency or complete loss of function associate to multisystem disorders in which eye lenses and skeletal and heart muscles might also be affected. This is the case of homozygous HSPB5 M1X and S21Afs*24 mutations, linked to infantile hypertonic myofibrillar myopathy (MFM), a severe and rare form of myopathy, often accompanied by cardiomyopathy and cataracts (Del Bigio et al., 2011; Ma et al., 2019; Lu et al., 2021). The HSPB5 M1X mutation determines the loss of the ATG starting codon and it likely associates with loss of HSPB5 protein. As mentioned, HSPB5/HSPB2 DKO mice are vital and do not display lens or cardiac alterations (Brady et al., 2001). The HSPB5 S21Afs*24 mutation is predicted to cause the production of a short protein product, carrying only the first 21 AA of HSPB5 NTD, while a completely different C-terminus of 44 AA. This HSPB5 frameshift mutant seems to be still expressed, although it is thought that mRNA decay affects its expression. Indeed, HSPB5 frameshift mutant immunoreactivity has been observed in inclusions positive for myosin, myoglobin, and desmin in patients muscle specimens (Del Bigio et al., 2011). Very similar observations have been made for the HSPB5 S115Pfs*14 frameshift mutant identified in infantile MFM (Forrest et al., 2011). Also in this case, HSPB5 mutant abnormal distribution was observed in the patient muscle biopsies, although its protein levels were decreased in respect to the HSPB5 WT in samples from a healthy subject (Forrest et al., 2011). The molecular mechanism underlying HSPB5 S115Pfs*14 mutant pathogenicity was deeply investigated using patient-derived induced Pluripotent Stem Cells (iPSCs), showing that iPSC-derived skeletal myotubes and cardiomyocytes are devoid of the mutated HSPB5 protein product, despite the presence of its related mRNA transcript (Mitzelfelt et al., 2016). Interestingly, the lack of the HSPB5 mutant protein apparently was not the result of miRNA targeting, defective translation, or disposal through the degradative systems, while, when the HSPB5 S115Pfs*14 mutant was overexpressed, its aggregation was observed (Mitzelfelt et al., 2016). Similarly, other truncated or frameshift mutants of HSPB5 have been found in the insoluble protein fractions and were prone to aggregate when overexpressed in cell models (Berry et al., 2001; Selcen and Engel, 2003; Simon et al., 2007a; Hayes et al., 2008; Zhang et al., 2010; Bortolani et al., 2020). Finally, falling at the very CTD end, mutations causing the abrogation of the stop codon with the formation of an elongated C-terminus have been described. These include the HSPB5 A172Pfs*14, linked to a multisystem syndrome, consisting of congenital cataract, hypotonia and a slight delay in motor skills acquisition without cognitive impairments, and HSPB5 X176Wext*19, linked to cataracts and DCM (Marcos et al., 2020; Yinhui Yu et al., 2021). It is not yet known which are the functional consequences of the HSPB5 A172Pfs*14 CTD modification, but, the presence of non-polar AA and the absence of the charged ones suggest a strong impact on protein solubility and functionality (Marcos et al., 2020). Similarly, the elongated tract at the CTD of HSPB5 X176Wext*19 has been predicted to cause HSPB5 CTD folding in a new α-helix and random-coil structure and its aggregation has been observed in cardiomyocytes from patients-explanted heart (van der Smagt et al., 2014; Yinhui Yu et al., 2021).

## 6 HSPB6

HSPB6 is a cytoplasmic protein of 17 kDa constitutively expressed in a wide variety of tissues, but predominantly in smooth, cardiac and skeletal muscle (Kato et al., 1994; Brophy et al., 1999; Fan and Kranias, 2011). HSPB6 mainly forms homodimers and tetramers in solution (van de Klundert et al., 1998; Bukach et al., 2004; Weeks et al., 2014; Delbecq et al., 2015). *In vitro*, HSPB6 has been found to interact with HSPB5, HSPB1 and HSPB7 (Bukach et al., 2009; Heirbaut et al., 2016; Heirbaut et al., 2017; Mymrikov et al., 2020; Shatov et al., 2020; Muranova et al., 2021). HSPB6 can also bind the co-chaperone BAG3 (Fuchs et al., 2009; Shemetov and Gusev, 2011; Morelli et al., 2017a; Rauch et al., 2017). HSPB6 possesses a RRXS motif in its NTD, which represents a consensus motif for the protein kinases PKA, PKG and PKD1. Indeed, HSPB6 serine-16 (S16) phosphorylation modulates protein-protein interaction and enhances its activity (Beall et al., 1999; Rembold et al., 2000; Sin and Baillie, 2015). For instance, it has been shown that phosphorylation promotes HSPB6 oligomers disassembly (Brophy et al., 1999), increases its interaction with Aβ, thus reducing or preventing Aβ-related toxicity (Cameron et al., 2014), and potentiates its antiapoptotic activity (Edwards et al., 2012). However, the major role of phosphorylated HSPB6 is related to cytoskeletal dynamics during muscle relaxation. Additionally, HSPB6 can undergo acetylation at lysine residues of its CTD (e.g., Lys160), which is reversed by KDAC8/HDAC8 activity (Chen et al., 2013). Both phosphorylation and acetylation are thought to mediate smooth muscle relaxation, as reported in studies conducted in airway and myometrial smooth muscles cells (Komalavilas et al., 2008; Tyson et al., 2008; Ba et al., 2009; Karolczak-Bayatti et al., 2011; Chen et al., 2013). It has been shown that HSPB6 acetylation and phosphorylation can occur together in response to increased PKA activity (Karolczak-Bayatti et al., 2011). Upon phosphorylation, HSPB6 is able to interact with the 14-3-3 protein, releasing the actin-binding protein cofilin, which, once dephosphorylated, favours actin depolymerization, thus muscle cells relaxation (Karolczak-Bayatti et al., 2011). However, given the weak interaction between 14-3-3 protein and cofilin, it is likely that other factors participate in cofilin dephosphorylation and depolymerizing activity (Sudnitsyna et al., 2012). Other mechanisms hypothesized to mediate muscle cell relaxation suggest that phosphorylated HSPB6 can directly inhibit myosin binding to actin filaments, as supported by the presence of a short motif highly homologous to troponin I inhibitory region in HSPB6 sequence (Rembold et al., 2000; Ba et al., 2009). However, *in vitro* analyses demonstrated that HSPB6 is unable to directly bind actin fibres (Bukach et al., 2005). Beside its role in smooth muscle relaxation, an HSPB6 protective activity is observed in cardiac cells. For instance, HSPB6 overexpression in rat cardiomyocytes determined an increase in cell viability and HSPB6 cardiac-specific TG mice showed an enhanced functional recovery and attenuated cardiac damage after I/R (Fan et al., 2005; Islamovic et al., 2007). Cardioprotection seems to rely on HSPB6 antiapoptotic activity in inhibiting pro-apoptotic factor Bax translocation to mitochondria and subsequent caspase-3 activation (Fan et al., 2005). Also in this case, phosphorylation promotes HSPB6 activity, since a phosphomimic HSPB6 mutant (S16D) increases the protection against apoptosis, while the phospho-null S16A mutant shows an attenuated protective activity (Fan et al., 2004; Qian et al., 2009). HSPB6 displays another cardiovascular protective activity, that relies on its secretion from cells. Indeed, plasma HSPB6, secreted within exosomes by cardiomyocytes and artery walls, can inhibit thrombin-mediated platelet aggregation by blocking calcium influx and favours angiogenesis (Niwa et al., 2000; Kozawa et al., 2002; McLemore et al., 2004; Zhang et al., 2012). The role of HSPB6 in the nervous system has also been investigated. HSPB6 mRNA and protein have been detected in the nervous system, and HSPB6 expression increases both during development and upon oxidative or hyperosmotic stress (Verschuure et al., 2003; Bartelt-Kirbach and Golenhofen, 2014). Similarly to other HSPBs, HSPB6 exerts an antiaggregant activity towards several misfolded and aggregating proteins, such as SNCA and Aβ, and it is found associated to AD plaques (Lee et al., 2005; Wilhelmus et al., 2006b; Bruinsma et al., 2011; Cameron et al., 2014). HSPB6 has been found in astrocytes of preactive and active lesions in autoptic samples from patients affected by X-linked adrenoleukodystrophy (Görtz et al., 2018) and in active lesion of subjects affected by Multiple Sclerosis (Peferoen et al., 2015). Of note, HSPB6 immunoreactivity has been described specifically in astrocytes surrounding areas of tissue damage in the lateral tracts of ALS patients, while no differences in HSPB6 staining were observed in neuronal cells of the spinal cord between patients and healthy subjects (Gorter et al., 2019). Although these observations suggest that HSPB6 exerts a role in the nervous system, such as the other HSPBs, its activity in neuronal cells is not well defined. *In vitro* studies evidenced its ability to interact with NF and to inhibit their oligomerization, but with a weaker activity in respect to other HSPBs (Nefedova et al., 2017). Neuroprotection was also investigated in epilepsy: upon pharmacologically induced seizures, rats showed HSPB6 upregulation and phosphorylation in the hippocampal neurons in concert with PKA pathway activation, while abrogation of PKA pathway resulted in increased apoptosis and pro-inflammatory factors (Qi et al., 2019).

Two mutations in HSPB6 have been reported in patients affected by DCM (Nicolaou et al., 2008; Liu et al., 2018a). HSPB6 P20L substitution has been described as a rare variant both in cardiomyopathy patients and in healthy subjects (Nicolaou et al., 2008). Nevertheless, rat cardiomyocytes overexpressing the HSPB6 WT or the P20L variant showed neither differences in HSPB6 protein levels nor variations in apoptosis in physiological conditions. However, when cells were challenged through I/R simulated conditions, the antiapoptotic activity of HSPB6 was completely abolished (Nicolaou et al., 2008). The S10F HSPB6 mutant has been extensively studied in a cardiac-selective TG mouse model (Liu et al., 2018b); these mice, with aging, displayed a pathologic phenotype with deterioration of cardiac functionality, progressive cardiac dilation and fibrosis, resulting in a halved life span. TG HSPB6 S10F mice cardiomyocytes displayed increased apoptotic events correlated to increased caspase-3 activation. Of note, TG HSPB6 S10F mice cardiomyocytes were characterized by a decreased autophagic flux, as demonstrated by a lesser conversion of the autophagic adaptor LC3-I to II and diminished levels of the autophagy regulator beclin-1. Notably, while HSPB6 WT can interact with beclin-1, displacing the beclin-1 suppressor Bcl-2, thus promoting autophagy, the HSPB6 S10F mutant loses this ability and favours beclin-1 ubiquitination and proteasomal degradation. In TG mice, beclin-1 instability ultimately resulted in loss of autophagic power and increased apoptosis, while its exogenous overexpression reverted the phenotype (Liu et al., 2018b). Noteworthily, cardiac phenotypes and molecular alterations have been observed only in TG HSPB6 S10F male mice, while minor changes affected the females under unstressed conditions, suggesting that HSPB6 S10F pathogenicity is subjected to gender differences (Liu et al., 2018a). Additional studies revealed that female TG HSPB6 S10F mice were unable to cope with pregnancy-associated cardiac stress. This resulted in cardiac dysfunction and remodelling and decreased survival after pregnancy, suggesting that HSPB6 S10F mutation carriers might be subjected to develop peripartum cardiomyopathy (Liu et al., 2018a). Structural analyses conducted *in vitro* showed that the HSPB6 P20L substitution affects the structure of HSPB6, by decreasing the β-sheet content toward an increase in α-helical or unordered structures (Nicolaou et al., 2008). Both HSPB6 P20L and S10F mutants were less resistant to heat-induced denaturation and aggregation, and more prone to oligomerization under crowded environment compared to the HSPB6 WT (Shatov and Gusev, 2020). Instead, no differences in HSPB6 hetero-oligomerization with HSPB1 and HSPB5 were observed (Shatov and Gusev, 2020). Finally, while the S10F mutant did not show variation in phosphorylation typical of the HSPB6 WT, the P20L mutant was characterized by an increased phosphorylation rate (Shatov and Gusev, 2020).

## 7 HSPB7

HSPB7 is a 19 kDa protein also known as cardiovascular HSP (cvHSP) (Krief et al., 1999). Three isoforms have been described as a result of mRNA alternative splicing: isoforms 1 and 2 correspond to two proteins of 170 and 175 AA, respectively, which differ for the presence of a AHPTA pentapeptide in the NTD; isoform 3 corresponds to a 68 AA-long protein (Krief et al., 1999). Beside the ACD, HSPB7 possesses a serine-rich stretch at position 17–29 in the NTD (Wu et al., 2019). Studies on HSPB7 homo- and hetero-interactions are conflicting (Golenhofen et al., 2004; Vos et al., 2010; Yang et al., 2011; Lin et al., 2014; Mymrikov et al., 2017). More recent *in vitro* evidence showed that HSPB7 forms mainly 600 kDa large oligomers and a small fraction of 36 kDa oligomers, and that the equilibrium between the two oligomeric states can be influenced by oxidation (Muranova et al., 2021). Indeed, HSPB7, like HSPB1, possesses a cysteine residue (C126) in the ACD, responsible of disulphide-crosslinked dimers formation and shifting of the oligomeric state towards large oligomers formation, upon mild oxidative conditions (Muranova et al., 2021). HSPB7 oligomerization is also affected by the NTD, since its deletion associates with small-size oligomers (Muranova et al., 2021). HSPB7 hetero-oligomerization has been described with HSPB6 and HSPB8 (Sun et al., 2004; Muranova et al., 2021). The interaction of HSPB7 with BAG3 is still controversial, but most studies support that HSPB7 is not a BAG3 partner and that its activities are BAG3-independent (Vos et al., 2010; Morelli et al., 2017a; Esslinger et al., 2017; Fang et al., 2019). Interestingly, the NTD of HSPB7 drives its localization into SC35 nuclear speckles (nuclear structures enriched in pre-mRNA and factors involved in splicing) where it appears to be a resident protein, but with unknown functions rather than substrates chaperoning (Vos et al., 2009). Moreover, the HSPB7 NTD mediates the HSPB7 anti-aggregating and pro-degradative activities against misfolded proteins involved in neurodegenerative diseases (NDs) via autophagy (Vos et al., 2010; Minoia et al., 2014; Wu et al., 2019). However, HSPB7 function seems not to be fundamental in NDs since its expression in the nervous system is physiologically low. Notably, even if HSPB7 mRNA has been detected in most tissues, HSPB7 protein has been observed only in myogenic tissues, such as in heart where its mRNA accounts for the 0.3% of total mRNA, but also in skeletal muscle and adipose tissue (Krief et al., 1999). In myogenic tissues, HSPB7 exerts a surveillance function on the cytoskeleton, by interacting with structural components, such as actin, filamin C and titin (Juo et al., 2016; Liao et al., 2017; Wu et al., 2017; Mercer et al., 2018). It has been postulated that HSPB7 might be considered a patho-biochemical indicator of muscle damage in dystrophinopathy. Indeed, mouse models of X-linked muscular dystrophy show higher levels of HSPB7 in respect to controls, which increase with aging, correlating with muscle degeneration. The increase of HSPB7 is thought to be a protective response to muscle fibres disintegration, in order to counteract the accumulation of damaged substrates and favour cytoskeletal repair (Doran et al., 2006; Doran et al., 2009; Lewis et al., 2009). HSPB7 upregulation, together with other HSPBs, has been also observed in skeletal muscle fibres of aging rats, in respect to young controls, supporting again a physiological role of HSPB7 in muscle structural maintenance and suggesting that strategies to induce its expression might be relevant to counteract aging-associated sarcopenia (Doran et al., 2007). HSPB7 has been also indicated as a candidate gene involved in heart development and cardiac failure (Villard et al., 2011; Aung et al., 2019). GWAS and EWAS defined HSPB7 gene *locus* as a risk factor to develop DCM or heart failure (Cappola et al., 2010; Stark et al., 2010; Villard et al., 2011; Garnier et al., 2015; Wang et al., 2016; Esslinger et al., 2017; Garnier et al., 2021). HSPB7 has also been detected in plasma, with HSPB7 plasmatic concentrations apparently higher in subjects and mouse models of myocardial infarction from 3 up to 24 h after the onset of symptoms (Chiu et al., 2012). HSPB7 plasma levels increase has been also described in early stages of heart failure in mouse models, suggesting that HSPB7 could be an early biomarker of heart damage (Rüdebusch et al., 2017). Also, HSPB7 variants have been found in sporadic cardiomyopathy (Matkovich et al., 2010). Recently, a single nucleotide polymorphism has been associated with obesity and higher fat intake, suggesting other mechanisms through which HSPB7 might be involved in cardiovascular disorders (Pavlová et al., 2018). Nevertheless, all the variants identified so far in humans are single nucleotide polymorphisms located in non-coding regions of the HSPB7 gene or are synonym mutations, causing no changes in the HSPB7 protein sequence, thus into its structure (Matkovich et al., 2010). Although HSPB7 mutations have not yet been identified in humans, a missense mutation has been recently described in cattle and associated with heat tolerance, whereas animal models mimicking HSPB7 deficiency have been generated to rule out the functions of HSPB7 in development and tissues maintenance (Zeng et al., 2019). These studies unravelled a dual role of HSPB7 in muscle development and maintenance, which agrees with the dynamic changes in HSPB7 localization in cells, mainly in mice cardiomyocytes, from early stages of embryonic life through adulthood (Liao et al., 2017; Wu et al., 2017). For instance, HSPB7 Knock-down (KD) in Zebrafish impacts on the development of left-right asymmetry of the heart, leading to morphologic defects (Lahvic et al., 2013; Rosenfeld et al., 2013). Consistent with the role of HSPB7 in development, both global and cardiac-selective KO in mice are lethal at the embryonic stage, with embryos characterized by smaller hearts, thinner left ventricular walls and features indicative of heart failure. Analyses of cardiomyocytes in HSPB7 KO mice revealed sarcomere disorganization, with longer thin filaments and the formation of abnormal actin filaments bundles containing Z-line components, such as α-actinin, but devoid of contractile components (Liao et al., 2017; Wu et al., 2017; Mercer et al., 2018). On the other hand, animal models from other studies allowed to investigate the role of HSPB7 in adult cells. Indeed, a HSPB7 KD Zebrafish model did not show severe developmental defects, but adult fish were affected by mild cardiac pathology with cardiomegaly and a higher rate of mortality upon exercise (Mercer et al., 2018). Consistently, HSPB7 conditional KO in mice determined cardiomyopathy and sudden death from arrythmia, which seem related to intercalated discs disruption, sarcomeres defective organization and filamin C aggregates observed in cardiac tissue (Liao et al., 2017). Filamin C aggregation has been observed in skeletal muscle-specific KO mice which developed a progressive myopathy phenotype, with loss of muscle fibres, defects in muscle morphology and increased mortality (Juo et al., 2016). Conversely, HSPB7 depletion in skeletal muscle cells indicated that HSPB7 is apparently not required during embryonic development (Juo et al., 2016). Finally, HSPB7 is expressed in adipocytes where it may have a role during differentiation (Imran et al., 2017; Jin et al., 2020; Rahman and Kim, 2020).

## 8 HSPB8

HSPB8 is a 22 kDa protein ubiquitously expressed, especially in cardiac and skeletal muscles. Although HSPB8 interaction with the others HSPBs has been reported, it is found mainly as a monomer and homodimer, interacting with the co-chaperone BAG3, with whom it forms a 2:1 stoichiometric complex through two BAG3 Isoleucine-Proline-Valine (IPV) domains (Kim et al., 2004; Sun et al., 2004; Fontaine et al., 2005; Fuchs et al., 2009; Shatov et al., 2021). The dependency of HSPB8 on BAG3 is supported not only by the fact that HSPB8 stability depends on BAG3 interaction, but also because HSPB8 has the highest affinity to BAG3 in respect to other BAG3-binding HSPBs (Carra et al., 2008a; Morelli et al., 2017a). In concert with BAG3, HSPB8 takes part in the Chaperone-Assisted Selective Autophagy (CASA), a selective autophagy pathway that mediates the removal of damaged and misfolded substrates (Carra et al., 2008b; Arndt et al., 2010). The CASA is mediated by a heteromeric complex formed by the associated proteins HSPB8 and BAG3 bound to the chaperone HSP70/HSPA and the E3 ubiquitin ligase carboxy-terminus of HSC70 interacting protein (CHIP/STUB1). HSPB8 activity consists in the recognition of substrates, and, by contacting BAG3, it helps in the HSP70/HSPA-driven refolding process of the substrate or in its degradation through autophagy (Gamerdinger et al., 2009; Gamerdinger et al., 2011; Rusmini et al., 2017). The role of BAG3 and HSPB8 interaction in the removal of misfolded proteins through autophagy was firstly observed in cells overexpressing proteins with elongated polyQ tracts, such as polyQ-HTT (Carra et al., 2008a). However, the first description of CASA complex composition and activity was reported in skeletal muscle cells by Arndt et al. (2010), who demonstrated the importance of the CASA complex in favouring the disposal of damaged components of the Z-disc structures, such as filamin, in response to its tension-induced unfolding upon mechanical stress (Ulbricht et al., 2013). CASA activity has been also demonstrated to be very efficient against mutated or aberrant protein products prone to misfold and found in inclusions in several NDs (Carra et al., 2005; Wilhelmus et al., 2006a; Carra et al., 2008a; Carra et al., 2008b; Crippa et al., 2010; Rusmini et al., 2013; Crippa et al., 2016; Cao et al., 2017; Cicardi et al., 2018; Cristofani et al., 2018; Cicardi et al., 2019). HSPB8 roles in cell homeostasis also comprise granulostasis, which is the process of stress granules (SGs) quality control in their composition and dynamics. In this context, HSPB8 is recruited to SGs where it acts as holdase, by preventing the irreversible aggregation of misfolded substrates and defective ribosomal products into SGs, and recruits BAG3-HSP70/HSPA machinery, for subsequent substrates disposal (Ganassi et al., 2016; Mateju et al., 2017). Also, HSPB8 promotes eukaryotic initiation factor-2α (eIF2α) phosphorylation to shut down translation of unnecessary proteins upon proteotoxicity (Carra et al., 2009). Recently, it has been shown that HSPB8 and BAG3 participate in the cytosolic unfolded protein response (cUPR), an integrated stress response pathway that mediates protein translation shutdown and degradation in response to proteotoxicity, acting on eIF2α kinase heme-regulated inhibitor (HRI) and eIF2α (Abdel-Nour et al., 2019; Mukherjee et al., 2021). HSPB8 is induced by different cell stresses that share the proteostasis-challenging feature, such as degradative systems blockade, but also sodium-arsenite, oxidative and osmotic stresses (Yew et al., 2005; Wilhelmus et al., 2006a; Bartelt-Kirbach and Golenhofen, 2014). Beside proteotoxic stress response, HSPB8 and BAG3 play an active role in cell division, by assuring actin structures homeostasis and dynamics during mitosis and cytokinesis (Fuchs et al., 2015; Varlet et al., 2017).

HSPB8 mutations are causative of dHMNs, CMT2 and myopathies, characterized by high variability in onset and progression. Noteworthily, BAG3 mutations are also associated with similar, even if often more severe, phenotypes (Selcen et al., 2009; Jaffer et al., 2012; Lee et al., 2012; Semmler et al., 2014; Kostera-Pruszczyk et al., 2015; Meister-Broekema et al., 2018; Shy et al., 2018). The most frequent encountered HSPB8 mutation consists in the substitution of the conserved lysine141 residue (K141E/N/T/M) (Irobi et al., 2004; Tang et al., 2005; Nakhro et al., 2013; Echaniz-Laguna et al., 2017a). HSPB8 K141 mutations have been mainly described in patients suffering from neuropathy, but myopathy with neurogenic involvement has been also linked to K141E mutation (Ghaoui et al., 2016). This conserved residue falls in the ACD, suggesting a role in dimerization. Noteworthily, HSPB8 K141E/N are not only still able to dimerize but also show an increased interaction with the HSPB8 WT (Fontaine et al., 2006). Instead, data on HSPB8 K141 mutants interaction with BAG3 are controversial, with some reports showing no changes in interaction, while others a decreased or increased interaction (Crippa et al., 2010; Shemetov and Gusev, 2011; Echaniz-Laguna et al., 2017a). Nevertheless, functional analyses *in vitro* and in cells showed a loss of chaperone activity and pro-degradative function of HSPB8 K141E/N toward certain protein models and misfolded substrates of CASA complex (Carra et al., 2005; Kim et al., 2006; Crippa et al., 2010). HSPB8 K141E/N loss of activity might be related to its own aggregation (Irobi et al., 2004; Irobi et al., 2012; Sanbe et al., 2013; Yang et al., 2017). HSPB8 K141E/N mutations have been extensively studied in patient biopsies, cells, and animal models. A first observation is that MNs are the primary target of HSPB8 K141E/N mutations as only primary MNs and N2a cell models show important neurite degeneration, while primary sensory neurons are only mildly affected and primary cortical neurons do not show signs of neurodegeneration (Irobi et al., 2010). Animal models recapitulate quite well the human phenotype. For instance, homozygous KI mice overexpressing HSPB8 K141N mutant showed MN pathology with progressive axonal degeneration and muscle atrophy, abnormal and degenerating organelles and accumulating mitochondria, which suggest an impaired autophagic flux. Indeed, sciatic nerve of pre-symptomatic mice showed low autophagy potential while post-symptomatic mice displayed HSPB8 aggregation. Moreover, homozygous KI mice manifested MFM signs with Z-disc disorganization, likely independent from the neuronal pathology, and characterized by aggregating HSPB8, HSPB5, desmin, and by the presence of rimmed vacuoles. Instead, the heterozygous model did not display signs of MNs or muscle dysfunction, but accumulating abnormal mitochondria were observed in muscle (Bouhy et al., 2018). Fly models overexpressing the HSPB8 ortholog Hsp67Bc carrying the R126E or R126N mutations, which correspond to human HSPB8 K141E or K141N mutants, showed normal muscle performance, but had histopathological hallmarks of muscle dysfunction, such as myofibrillar disorganization (Carra et al., 2010; Jabłońska et al., 2018). Mitochondria abnormalities observed in mice, fly models and patient fibroblasts, suggest that these organelles are a target of HSPB8 mutant-mediated cytotoxicity (Irobi et al., 2012). Of note, HSPB8 exerts a protective activity upon oxidative stress by inhibiting ROS formation and mitochondrial apoptotic pathway, while HSPB8 K141N mutant expression associates with higher ROS levels and reduced response to oxidative stress (Jo et al., 2017; Yang et al., 2017; Yu et al., 2019). In addition, HSPB8-BAG3 complex regulates the antioxidant axis of Kelch-like ECH-associated protein 1-nuclear factor (erythroid-derived 2)-like 2, Keap1-Nrf2. For instance, cells expressing HSPB8 K141N mutant showed impaired Nrf2 nuclear translocation which was reverted by antioxidants, and similar alterations have been observed in cells expressing the myopathy-related BAG3 P209L mutant (Yang et al., 2017; Guilbert et al., 2018). HSPB8 is also involved in RNA metabolism: HSPB8 can bind the RNA helicase DDX20, which is a component of Survival Motor Neuron (SMN) and small nuclear ribonucleoproteins (snRNP) complexes, and HSPB8 K141 mutants show alteration of DDX20 binding (Sun et al., 2010). Moreover, a deregulation in the RNA binding protein (RBP) TDP-43 activity has been observed in dHMN and MFM patients carrying the K141E mutation; in particular, alteration of splicing of TDP-43 target genes and decreased mRNA levels of TDP-43 correlating to HSPB8 aggregation were observed in patients muscles (Cortese et al., 2018). In summary, mutations in HSPB8 K141 hotspot are characterized both by a loss of protective function, and by a likely gain of toxic function. Recently, four different frameshift mutations have been linked to autosomal dominant myopathies: the HSPB8 P173Sfs*43, T176Wfs*38, Q170Gfs*45 and T194Sfs*23 (Echaniz-Laguna et al., 2017b; Al-Tahan et al., 2019; Nicolau et al., 2020; Inoue-Shibui et al., 2021). Although these mutations are often causative of primary myopathies, neurogenic involvement can be observed (Ghaoui et al., 2016). Histopathological features associated with the Q170Gfs*45 mutation consist in signs of MFM with protein aggregates formed by structural components such as desmin, dystrophin, myotilin and members of the proteostasis network, such as HSPB8 itself, BAG3, HSPB5, ubiquitin, but also TDP-43, and rimmed vacuoles (Echaniz-Laguna et al., 2017b). Similar histopathological signs were observed in T194Sfs*23 mutation carriers, with myofibrillar aggregates and rimmed vacuoles (Nicolau et al., 2020). Strikingly, all these frameshift mutations fall in the CTD of HSPB8 and cause the production of HSPB8 proteins with a variably modified CTD and a common extended C-terminal tail, resulting from the use of an alternative downstream stop codon. Evaluation of the extended C-terminal tail by *in silico* tools revealed a decreased solubility and an increased aggregation propensity of a tract corresponding to the ILV tripeptide, which resembles the I/V-X-I/V domain shared by several HSPBs, but not present in HSPB8 (Inoue-Shibui et al., 2021). Interestingly, previous biochemical studies investigating HSPBs interaction with BAG3 showed that the introduction of the CTD of HSPB1, which presents a I/V-X-I/V motif, into HSPB8, decreased HSPB8 solubility, suggesting a deleterious effect of I/V-X-I/V motif-like sequence on HSPB8 dynamics (Fuchs et al., 2009). In addition, I/V-X-I/V motif mutations in HSPB1, and similarly in BAG3, are linked to neuromyopathies, strengthening the idea of a role of the new ILV motif in HSPB8 frameshift mutants-related diseases (Selcen et al., 2009; Jaffer et al., 2012; Semmler et al., 2014; Shy et al., 2018; Adriaenssens et al., 2020). However, the effects of the novel HSPB8 frameshift mutations are not yet clear. It has been suggested that HSPB8 frameshift mutations cause HSPB8 haploinsufficiency, since no extended HSPB8 species have been detected and halved HSPB8 protein levels were observed; this is likely related to a rapid degradation of mutant HSPB8, which has been reported in muscle and fibroblast cells (Echaniz-Laguna et al., 2017b). Noteworthily, HSPB8 KO mice do not manifest any myopathic phenotype, suggesting that a simple loss of function of HSPB8 frameshift mutants is not sufficient to induce muscle diseases (Bouhy et al., 2018). Indeed, it is likely that HSPB8 frameshift mutants cause an impairment in CASA pathway which determines an insufficient clearance of aggregates, as suggested by the accumulation of the autophagic markers LC3 and SQSTM1/p62 in patient fibroblasts; alternatively, it is possible that HSPB8 mutants are prone to form aggregates themselves (Al-Tahan et al., 2019). Moreover, impairments of RNA metabolism and SGs formation are suggested as other pathological mechanisms of HSPB8 frameshift mutations. Indeed, HSPB8 T194Sfs*23 mutation was associated with abnormal punctate distribution of TIA-1, a component of SGs (Nicolau et al., 2020). Interestingly, HSPB8 mutations have not been correlated with cardiomyopathies, unlike BAG3 substitutions. However, TG mice overexpressing HSPB8 K141N in cardiac tissue show aggregating HSPB8 and mitochondrial dysfunction in cardiomyocytes and develop signs of cardiomyopathy at the age of 6 months (Sanbe et al., 2013), while KO mice show cardiac defects with aging (Wu et al., 2021). HSPB8 variants have been also described in cancer (e.g., W51C and P173H (Gober et al., 2003; Smith et al., 2011)), in which HSPB8 role has been recently extensively reviewed (Cristofani et al., 2021).

## 9 HSPB9 and HSPB10

Little is known about HSPB9 and HSPB10, which are testis-specific HSPBs. HSPB9 has a MW of 17.5 kDa and, among the orthologous HSPBs, its protein sequence is the most divergent between mouse and human (Kappé et al., 2001). HSPB9 expression is confined in testis germ cells and varies during spermatogenesis. HSPB9 has been also detected in tumors, suggesting HSPB9 as a candidate Cancer/Testis Antigen (de Wit et al., 2004). Studies on HSPB9 oligomerization are missing, but yeast two-hybrid screening revealed its interaction through the CTD with the t-complex testis expressed protein-1, a light chain subunit of dynein, which also follows a similar expression pattern in testis and cancers (de Wit et al., 2004). To our knowledge, the roles of HSPB9 have not yet been investigated neither in testis nor in tumorigenesis and mutations have not been identified. HSPB10 association with HSPBs dates to 2003, when two independent groups identified the Outer Dense Fibre protein 1 (ODF1) as the last member of HSPBs family (Fontaine et al., 2003; Kappé et al., 2003). HSPB10 protein is the largest member of the family, with a MW of 28.4 kDa and a highly extended NTD. HSPB10 expression is confined to testis, especially in spermatids, where it takes part in the formation of the Outer Dense Fibres (ODF), cytoskeletal accessory structures of sperm tails fundamental for elastic recoil (Baltz et al., 1990; Gastmann et al., 1993; Burmester and Hoyer-Fender, 1996). Recently, HSPB10 protein has been detected also in kidney collecting ducts, suggesting that HSPB10 might play a structural role in specialized structures, such as cilia and flagella (Cabrillana et al., 2019). Beside the ACD, HSPB10 structure is characterized by the presence of several C-X-P repeats in the CTD and a putative leucine-zipper domain in the NTD (Shao and van der Hoorn, 1996). Yeast two-hybrid screening studies revealed that HSPB10 might self-interact through its putative leucine-zipper domain and that it multimerizes *in vitro* at high concentrations (Shao and van der Hoorn, 1996). Also, the leucine-zipper motif of HSPB10 has been found to mediate interactions with other constituents of the ODF structure, such as ODF2, the sperm-associated antigens SPAG4, an axoneme-binding protein, and SPAG5, a component of the mitotic spindle, and the kinesin light chain 3 (Petersen et al., 1999; Shao et al., 1999; Shao et al., 2001; Bhullar et al., 2003; Fitzgerald et al., 2006). Instead, the C-X-P rich domain was found to mediate HSPB10 interaction with ODF1-interacting protein (OIP1), a member of the Really Interesting New Gene finger family and a putative E3 ubiquitin protein ligase (Zarsky et al., 2003). It has been hypothesized that this interaction, which is stronger when HSPB10 is phosphorylated at serine (S196), might favour HSPB10 degradation, thus promoting ODF complex disassembly and sperm tail detachment after fertilization (Rosales et al., 2007). Notably, a high content of cysteines characterizes the HSPB10 protein sequence, and it has been proposed that thiol groups redox status changes during spermatozoa maturation and its alteration impacts on spermatozoa motility (Kappé et al., 2003; Dias et al., 2014; Cabrillana et al., 2017). HSPB10 role in diseases has been strictly investigated in the field of male fertility, even if no HSPB10 pathogenic mutation has yet been identified in humans. Indeed, only one homozygous variant, consisting in a 27-bp deletion in the C-X-P repeat-rich CTD, has been reported in literature so far, but it resulted not being associated to any pathogenic phenotype (Hofferbert et al., 1993). On the other hand, a reduction of HSPB10 levels has been observed within the gametes in a small cohort of infertile idiopathic male patients in respect to fertile donors, suggesting HSPB10 as fertility biomarker (Hetherington et al., 2016). Interestingly, spermatozoa of infertile subjects resulted vulnerable to decapitation after freeze-thawing stress induction (Hetherington et al., 2016). Mice showed a similar phenotype when HSPB10 was depleted (Kappé et al., 2003; Yang et al., 2012; Yang et al., 2014). Indeed, homozygous HSPB10-deficient mice, which are infertile, are characterized by defects in head-to-tail linkage in sperm, with decapitated spermatozoa displaying mitochondrial disorganization (Yang et al., 2012). Notably, heterozygous male mice, which showed halved levels of HSPB10, were fertile or subfertile, but still characterized by a weakening of the sperm head-to-tail coupling and decreased sperm motility (Yang et al., 2014). HSPB10 ectopic expression has also been found in some cancers, suggesting that, as HSPB9, it should be evaluated as a cancer biomarker (Ghafouri-Fard et al., 2010; Li et al., 2021).

## 10 Discussion

HSPBs are a subset of chaperones playing multiple roles aimed at maintaining cell homeostasis. HSPBs main roles are exploited through pathways related to the protein quality control (PQC) system and consist in recognition of damaged and misfolded proteins, protein aggregation prevention, cooperation with other chaperones and the degradative systems for protein substrates refolding or disposal.

Interestingly, the expression of some HSPBs is confined to specific tissues, while others are broadly expressed. However, although many HSPBs are ubiquitously expressed, some tissues strictly rely on HSPBs for the accomplishment of different physiological activities. For instance, both skeletal and cardiac muscle require structural integrity of the cytoskeletal components for cell contraction; neuronal cells cytoskeletal architecture must be preserved for axonal transport and communication with target cells; eye lenses fibres need to prevent crystallin proteins damage and aggregation to assure lens transparency. Overall, these cell types are characterized by an absent or low cell turnover (post-mitotic or with low mitotic index cells), which suggests that the maintenance of a healthy proteome is essential from early ages and during aging. Besides proteostasis, HSPBs modulate cell death. The redundance of HSPBs functions suggests that those tissues expressing several HSPBs might be able to obviate the absence of one HSPB through its replacement with another one. On the other hand, several studies suggest that HSPBs activities diverged to specialized functions that cannot be accomplished by the other members of the family. To mention few examples, it has been shown that HSPB2 plays a role in cells energetics and nuclear integrity; HSPB6 in cell muscle relaxation, HSPB8 in cell division, protein translation and SGs dynamics.

On these bases, it is expected that mutations in HSPBs affect those tissues in which HSPBs themselves are predominantly or exclusively expressed, giving rise to a clear clinical phenotype in affected patients. However, mutations in HSPBs with a pleiotropic activity might be related to different or multiple phenotypes. For instance, HSPB5 mutations have been linked to cataracts or cardiomyopathy or myopathy, but also to multiple system diseases in which all these pathological conditions are observed. Similarly, HSPB1 or HSPB8 have been described in neuropathies and/or myopathies. Moreover, different phenotypes can be linked to the same mutation, suggesting that other genetics, epigenetics, or exogenous factors have a role in predisposition to a certain disease. Importantly, most of the HSPBs mutations are dominantly inherited.

Of note, mutations in HSPBs have been identified along the whole structure. Mutations include missense, nonsense, and frameshift mutations that might affect HSPBs protein expression and stability, PTMs, dynamics in oligomerization and interaction with target proteins, thus altering HSPBs activity. While some HSPBs mutations have been deeply investigated *in vitro* and *in vivo*, others have not been subjected to a deep characterization and the number of mutations is still growing. Thus, the mechanisms of action of several mutants are not defined, yet.

Instead, other pathogenic mechanisms are quite well defined. One of the mechanisms at the basis of HSPBs-related diseases includes their complete deficiency. This is the case of nonsense or frameshift mutations that cause the complete loss of protein production or, as in the case of mutations falling in the NTD or ACD, severe modification of the protein itself. Several mutations that are associated with a complete loss of the HSPBs protein products cause the disease only in homozygosis, while heterozygous carriers are unaffected, suggesting that the WT allele is sufficient to accomplish its functions. Instead, haploinsufficiency mechanisms have been hypothesized in the case of frameshift mutations affecting HSPBs protein sequence to a lesser extent (e.g., mutations falling at the CTD). In this case, it has been suggested that decreased levels of a HSPB might be sufficient for disease manifestation. However, several studies also report that several HSPBs frameshift mutants, when expressed in cell models, are prone to aggregate. Indeed, HSPBs aggregation is observed for several HSPBs mutations, including also missense mutations, and represents another mechanism of pathogenicity. Protein aggregation is a hallmark of muscle and neuron diseases and associates with several intracellular alterations. Indeed, aberrant oligomeric structures and aggregates may impair the PQC system, by overwhelming it or by sequestering not only the other HSPBs, but also key factors involved in proteostasis. In addition, these toxic species directly affect cytoskeletal structures and, in the case of HSPBs mutants, co-segregate with structural components. Finally, other intracellular structures and organelles might be damaged by these toxic species. Overall, these alterations increase the vulnerability to cell death. Therapeutic strategies to counteract the onset or progression of HSPBs-related diseases should be developed based on the pathogenic mechanism (e.g., HSPB deficiency or aggregation) and on the cell types affected. For example, boosting the expression of other HSPBs has been hypothesized to be a possible therapeutic approach to replace a dysfunctional and mutated HSPB and to favour its disposal, in the case of its aggregation. Alternatively, strategies to revert the intracellular alterations caused by HSPBs mutations might be beneficial. For instance, drugs or approaches to counteract oxidative stress, cytoskeletal modifications or stress-response pathways that might lead to cell death have been also tested. Thus, mutations in HSPBs determine a loss of their functional activity, which can be accompanied by a gain of toxic functions and/or by a dominant action on the WT allele, underlying that mutated HSPBs pathogenicity manifests through multiple ways.

## Glossary

AA: Amino acid
ACD: α-Crystallin Domain
AD: Alzheimer's Disease
ALS: Amyotrophic Lateral Sclerosis
Aβ: Amyloid β
BAG3: Bcl-2 Associated Athanogene 3
CASA: Chaperone-Assisted Selective Autophagy
CHIP/STUB1: Carboxy-terminus of HSC70 interacting protein
CMT2: Charcot-Marie-Tooth type 2
CT51: Cancer/Testis Antigen 51
CTD: C-terminal Domain
cUPR: Cytosolic Unfolded Protein Response
dHMNII: distal Hereditary Motor Neuropathy type II
DKO: Double Knock-out
eIF2α: Eukaryotic initiation factor-2α
ER: Endoplasmic Reticulum
HRI: eIF2α kinase heme-regulated inhibitor
HSP70/HSPA: Heat Shock Protein 70
HTT: Huntingtin
I/R: Ischemia/Reperfusion
iPSCs: Induced Pluripotent Stem Cells
KD: Knock-down
Keap1: Kelch-like ECH-associated protein 1
KI: Knock-in
KO: Knock-out
LLPS: Liquid-Liquid Phase Separation
MFM: Myofibrillar Myopathy
MKBP: Myotonic dystrophy protein Kinase Binding Protein
MNs: Motoneurons
MW: Molecular Weight
NDs: Neurodegenerative Diseases
NF: Neurofilament
Nrf2: Nuclear factor (erythroid-derived 2)-like 2
NTD: N-terminal Domain
ODF1: Outer Dense Fibre protein 1
ODF2: Outer Dense Fibre protein 2
OIP1: ODF1-interacting protein
polyQ: Polyglutamine
PQC: Protein Quality Control
PTM: Post-translational modification
RBP: RNA binding protein
SBMA: Spinal and Bulbar Muscular Atrophy
SGs: Stress Granules
sHSPs or HSPBs: small Heat Shock Proteins or Heat Shock Proteins B
SMN: Survival Motor Neuron
SNCA: α-synuclein
snRNP: Small nuclear ribonucleoprotein
SOD1: Superoxide Dismutase 1
SPAG: Sperm-associated antigen
SQSTM1/p62: Sequestosome1/p62
TG: Transgenic
UPR: Unfolded Protein Response
UPS: Ubiquitin Proteasome System
WT: Wild-type
