# Supplementary material for: Insights on Human Small Heat Shock Proteins and Their Alterations in Diseases
Source: Front Mol Biosci. 2022 Feb 25;9:842149. doi: 10.3389/fmolb.2022.842149 (PMC8913478; doi:10.3389/fmolb.2022.842149)
Supplement: Supplementary file 1 [file DataSheet1.docx]

Supplementary Material

# Supplementary Table 1.

List of HSPBs mutations, inheritance, and associated diseases. D, Dominant; R, Recessive; N, Neuropathy, M, Myopathy; ALS-like, Amyotrophic Lateral Sclerosis-like phenotype; C, Cataract; CC, congenital cataract; CM, cardiomyopathy.

|  | Nucleotide | Protein | Inheritance | | Disease | References |
| --- | --- | --- | --- | --- | --- | --- |
| HSPB1 | c.19C>T / c.20C>G | p.P7S/R | | D | N | (Luigetti et al., 2016; Echaniz-Laguna et al., 2017a; Fortunato et al., 2017) |
|  | c.100G>A | p.G34R | | de novo | N | (Capponi et al., 2011) |
|  | c.116C>T | p.P39L | | D | N | (Houlden et al., 2008; Capponi et al., 2011; Echaniz-Laguna et al., 2017a; Rossor et al., 2017; Tanabe et al., 2018) |
|  | c.121G>A | p.E41K | | D | N | (Capponi et al., 2011) |
|  | c.158G>A | p.G53D | | R | N | (Echaniz-Laguna et al., 2017a) |
|  | c.165-171 dup | p.L58Afs*105 | | D | N | (DiVincenzo et al., 2014; Echaniz-Laguna et al., 2017a) |
|  | c.180dupC | p.A61Rfs*100 | | D | N | (Echaniz-Laguna et al., 2017a) |
|  | c.250G>C | p.G84R | | D/ de novo | N | (Houlden et al., 2008; James et al., 2008; Rossor et al., 2017) |
|  | c.257C>T | p.S86L | | R | N | (Scarlato et al., 2015) |
|  | c.295C>A | p.L99M | | R | N | (Rossor et al., 2017) |
|  | c.367A>T | p.K123X | | de novo | N | (Capponi et al., 2011; Tanabe et al., 2018) |
|  | c.379C>T / c.380G>T | p.R127W/L | | D | N | (Tang et al., 2005a; Dierick et al., 2008; Benedetti et al., 2010; Solla et al., 2010; Capponi et al., 2011; Ylikallio et al., 2015; Echaniz-Laguna et al., 2017a; Lorefice et al., 2017; Tanabe et al., 2018) |
|  | c.383A>G | p.Q128R | | D | N | (Echaniz-Laguna et al., 2017a) |
|  | c.387C>G | p.D129E | | D | N/M | (Lewis-Smith et al., 2016) |
|  | c.404C>T / c.404C>G / c.404C>A | p.S135F/C/Y | | D/ de novo | N | (Ismailov et al., 2001; Evgrafov et al., 2004; Houlden et al., 2008; Benedetti et al., 2010; Ylikallio et al., 2014; Echaniz-Laguna et al., 2017a; Rossor et al., 2017) |
|  | c.406C>T / c.407G>T / c.407G>C | p.R136W/L/H | | D | N | (Evgrafov et al., 2004; Capponi et al., 2011; Frasquet et al., 2021) |
|  | c.416C>T | p.T139M | | D | N | (Amornvit et al., 2017) |
|  | c.418C>G | p.R140G | | D/R | N/M | (Houlden et al., 2008; Bugiardini et al., 2017) |
|  | c.421A>C | p.K141Q | | D | N | (Ikeda et al., 2009; Maeda et al., 2014; Tanabe et al., 2018) |
|  | c.452C>T | p.T151I | | D/ de novo | N | (Evgrafov et al., 2004) |
|  | c.476-477 delCT | p.S158fs*200 *p.P159Rfs*42* | | D | N | (Mandich et al., 2010; Capponi et al., 2011) |
|  | c.490A>G | p.T164A | | D | N | (Lin et al., 2011) |
|  | c.505delA | p.M169Cfs*4 | | de novo | N | (Ylikallio et al., 2015) |
|  | c.523C>T | p.Q175X | | D | N | (Rossor et al., 2012) |
|  | c.539C>T | p.T180I | | D/ de novo | N | (Luigetti et al., 2010) |
|  | c.544C>G / c.545C>T / c.544C>T | p.P182A/L/S | | D/ de novo | N | (Evgrafov et al., 2004; Kijima et al., 2005; Rossor et al., 2017) |
|  | c.560C>T | p.S187L | | de novo | N | (Echaniz-Laguna et al., 2017a) |
|  | c.562C>T | p.R188W | | de novo | N | (Capponi et al., 2011) |
|  | c.570G>C | p.Q190H | | likely D | ALS-like | (Capponi et al., 2016b) |
|  | c.610dupG | p.A204Gfs*6 | | likely D | ALS-like | (Capponi et al., 2016b) |
| HSPB3 | c.18G>C | p.L6F | | D | N | (Yalcintepe et al., 2021) |
|  | c.21G>T | p.R7S | | D | N | (Kolb et al., 2010) |
|  | c.98dupC | p.L34Ffs*50 | | likely D | M | (Morelli et al., 2017) |
|  | c.347G>C | p.R116P | | D | M | (Morelli et al., 2017) |
|  | c.352T>C | p.Y118H | | D | N | (Nam et al., 2018) |
| HSPB4 | c.27G>A | p.W9X | | R | CC | (Pras et al., 2000) |
|  | c.34C>T / c.35G>T | p.R12C/L | | D | CC | (Hansen et al., 2007; Song et al., 2018) |
|  | c.61C>T / c.62G>A | p.R21C/W/L/Q | | D | CC | (Graw et al., 2006; Hansen et al., 2007; Laurie et al., 2013) |
|  | c.145C>T | p.R49C | | D | CC | (Mackay et al., 2003) |
|  | c.160C>T / c.161G>C / c.161G>T | p.R54C/P/L | | R/D | CC | (Khan et al., 2007; Devi et al., 2008; Su et al., 2012; Yang et al., 2013) |
|  | c.194G>A | p.R65Q | | likely D | Age-related C | (Patel et al., 2017) |
|  | c.213C>A | p.F71L | | R? | Age related C | (Bhagyalaxmi et al., 2009) |
|  | c.292G>A | p.G98R | | D | Age-related C/CC | (Santhiya et al., 2006) |
|  | c.346C>T / c.346C>G / c.347G>A | p.R116C/H | | D | CC | (Litt et al., 1998; Li et al., 2010; Sun et al., 2011) |
|  | c.344_352 del | p.116-118del | | likely D | CC | (Li et al., 2017) |
|  | c.346_348 delCGC | p.117delR | | D | CC | (Kong et al., 2015) |
|  | c.350_352 delGCT | p.R117H,Y118 del | | D | CC | (Sun et al., 2011) |
|  | c.356G>A | R119H | | likely D | Age-related C | (Patel et al., 2017) |
|  | c.416T>C | p.L139P | | D | CC | (Liang et al., 2015) |
|  | c.440delA | p.Q147Rfs*48 | | D | CC | (Javadiyan et al., 2017) |
|  | c.520T>C; c.521A>C | p.X174Qext*41; p.X174Sext*41 | | de novo, likely D | Severe eye defects | (Marakhonov et al., 2020) |
| HSPB5 | c.3G>A | p.M1X | | R | M | (Ma et al., 2019) |
|  | c.34C>T / c.31C>T / c.32G>A | p.R11C/H | | D | CC | (Chen et al., 2009; Jiao et al., 2015) |
|  | c.59C>G / c.58C>T | p.P20R/S | | D | CC | (Liu et al., 2006a; Xia et al., 2014) |
|  | c.60delC | p.S21Afs*24 | | R | M | (Del Bigio et al., 2011) |
|  | c.166C>T | p.R56W | | R | CC | (Safieh et al., 2009; Khan et al., 2010) |
|  | c.205C>T | p.R69C | | D | CC | (Sun et al., 2011) |
|  | c.326A>C / c.325G>C / c.326A>G | p.D109A/H/G | | D | M+C+CM | (Sacconi et al., 2012; Brodehl et al., 2017; Fichna et al., 2017; Potulska-Chromik et al., 2021) |
|  | c.343delT | p.S115Pfs*14 | | R | M | (Forrest et al., 2011) |
|  | c.358A>G | p.R120G | | D | M+ C+CM | (Vicart et al., 1998) |
|  | c.367C>T | p.R123W | | / | CM | (Maron et al., 2020) |
|  | c.418G>A | p.D140N | | D | CC | (Liu et al., 2006b) |
|  | c.450delA | p.K150Nfs*34 | | D | C | (Berry et al., 2001) |
|  | c.451C>T | p.Q151X | | D | M | (Bortolani et al., 2020) |
|  | c.464–465 delCT | p.P155Rfs*9 | | D | M+N | (Selcen and Engel, 2003) |
|  | c.460G>A | p.G154S | | D | CM/M | (Reilich et al., 2010) |
|  | c.470G>A | p.R157H | | D | CM | (Inagaki et al., 2006) |
|  | c.514G>A | p.A171T | | D | CC | (Devi et al., 2008) |
|  | c.514delG | p.A172Pfs*14 | | D | M+C | (Marcos et al., 2020) |
|  | c.527A>G | p.X176Wext*19 | | D | CM+C | (van der Smagt et al., 2014; Yu et al., 2021) |
| HSPB6 | c.29C>T | p.S10F | |  | CM | (Liu et al., 2018b) |
|  | c.59C>T | p.P20L | |  | CM | (Nicolaou et al., 2008) |
| HSPB8 | c.421A>G / c.423G>C / c.423G>T / c.422A>C / c.422A>T | p.K141E/N/T/M | | D | N/M | (Irobi et al., 2004; Tang et al., 2005b; Nakhro et al., 2013; Ghaoui et al., 2016; Echaniz-Laguna et al., 2017a) |
|  | c.269C>T | p.P90L | | de novo | N | (Echaniz-Laguna et al., 2017a) |
|  | c.413A>C | p.N138T | | D | N | (Echaniz-Laguna et al., 2017a) |
|  | c.515dupC | p.P173Sfs*43 | | D | N/M | (Ghaoui et al., 2016; Al-Tahan et al., 2019) |
|  | c.508_509 delCA | p.Q170Gfs*45 | | D | M | (Echaniz-Laguna et al., 2017b) |
|  | c.577_580 dupGTCA | p.T194Sfs*23 | | D | M | (Nicolau et al., 2020) |
|  | c.525_529 del | p.T176Wfs*38 | | D | M | (Inoue-Shibui et al., 2021) |

# Supplementary Table 2

List of HSPBs mutations and effects on their structure, properties, and functions.

|  | mutation | stability | | dimerization | | oligomers size / aggregation | hetero-oligomerization | proteostatic function | anti-apoptotic activity /  cell viability | Cytoskeleton alteration | animal models | Other features | References |
| --- | --- | --- | --- | --- | --- | --- | --- | --- | --- | --- | --- | --- | --- |
| HSPB1 | p.P7S/R | |  | |  |  |  |  |  | ↑ |  |  | (Echaniz-Laguna et al., 2017a) |
|  | p.G34R | |  | |  | ↑ |  | ↓ |  |  |  |  | (Muranova et al., 2015) |
|  | p.P39L | |  | |  | ↑ |  | ↓ | ↓ |  |  | impaired transport and activity of mitochondria | (Muranova et al., 2015; Kalmar et al., 2017) |
|  | p.E41K | |  | |  | ↑ |  | ↓ |  |  |  |  | (Muranova et al., 2015) |
|  | p.G53D | |  | |  |  |  |  |  | ↑ |  |  | (Echaniz-Laguna et al., 2017a) |
|  | p.L58Afs*105 | | ↓ | |  |  |  |  |  |  |  |  | (Echaniz-Laguna et al., 2017a) |
|  | p.A61Rfs*100 | | ↓ | |  |  |  |  |  |  |  |  | (Echaniz-Laguna et al., 2017a) |
|  | p.G84R | |  | |  | ↑ ; less stable | ↓ with HSPB6 | ↓ |  |  |  |  | (James et al., 2008; Nefedova et al., 2013b) |
|  | p.S86L | |  | |  |  |  |  |  |  |  |  |  |
|  | p.L99M | |  | |  | ↑ ; less stable | ↓ with HSPB6 | ↓ |  | = |  |  | (Nefedova et al., 2013b, 2017) |
|  | p.K123X | |  | |  |  |  |  |  |  |  |  |  |
|  | p.R127W/L | |  | | ↑/↓ |  |  | ↓ autophagy | ↓ | ↑ | mouse |  | (Almeida-Souza et al., 2010; Srivastava et al., 2012; Ylikallio et al., 2015; Haidar et al., 2019) |
|  | p.Q128R | |  | | = |  |  |  |  | ↑ |  |  | (Echaniz-Laguna et al., 2017a) |
|  | p.D129E | |  | |  |  |  |  |  |  |  |  |  |
|  | p.S135F/C/Y | |  | | ↓ | ↑ |  | ↓ autophagy | ↓ | ↑ | mouse / *fly* | impaired transport and activity of mitochondria | (Evgrafov et al., 2004; Almeida-Souza et al., 2010; d’Ydewalle et al., 2011; Holmgren et al., 2013; Lee et al., 2015; Kalmar et al., 2017; Haidar et al., 2019; Kang et al., 2020) |
|  | p.R136W/L/H | |  | | ↓ | ↑ |  |  |  | ↑ | mouse |  | (Almeida-Souza et al., 2010; Srivastava et al., 2012) |
|  | p.T139M | |  | |  |  |  | ↓ | ↓ |  |  |  | (Amornvit et al., 2017) |
|  | p.R140G | |  | |  | ↑ | ↓ with HSPB6 | ↓ | ↓ | = |  | impaired transport and activity of mitochondria | (Evgrafov et al., 2004; Nefedova et al., 2013a, 2017; Kalmar et al., 2017) |
|  | p.K141Q | |  | |  | ↑ |  | = |  | = |  |  | (Nefedova et al., 2013a, 2017) |
|  | p.T151I | |  | |  |  |  |  |  |  |  |  |  |
|  | p.S158fs*200 *p.P159Rfs*42* | |  | |  |  |  |  |  |  |  |  |  |
|  | p.T164A | |  | |  |  |  | = |  |  |  |  | (Chalova et al., 2014) |
|  | p.M169Cfs*4 | |  | | = |  |  | ↓ |  |  |  |  | (Ylikallio et al., 2015) |
|  | p.Q175X | |  | |  |  |  |  |  |  |  |  |  |
|  | p.T180I | |  | |  |  |  | = |  |  |  |  | (Chalova et al., 2014) |
|  | p.P182A/L/S | |  | |  | ↑ |  | ↓ autophagy |  | ↑ / = | mouse |  | (Evgrafov et al., 2004; Ackerley et al., 2006; Srivastava et al., 2012; Holmgren et al., 2013; Nefedova et al., 2017; Haidar et al., 2019) |
|  | p.S187L | |  | |  | ↑ |  |  |  |  |  |  | (Echaniz-Laguna et al., 2017a) |
|  | p.R188W | |  | |  |  |  | ↓ |  |  |  |  | (Chalova et al., 2014) |
|  | p.Q190H | |  | |  |  |  |  |  |  |  |  |  |
|  | p.A204Gfs*6 | | ↓ | | = |  |  | ↓ |  |  |  |  | (Capponi et al., 2016a) |
| HSPB3 | p.L6F | |  | |  |  |  |  |  |  |  |  | (Yalcintepe et al., 2021) |
|  | p.R7S | |  | |  |  | = with HSPB3 |  |  |  |  |  | (Clark et al., 2018) |
|  | p.L34Ffs*50 | | ↓ | |  |  |  |  |  |  |  | altered HSPB2-LLPS in nuclei | (Morelli et al., 2017) |
|  | p.R116P | |  | |  | ↑ | ↓ with HSPB3 |  |  |  |  | Intranuclear aggregation; altered HSPB2-LLPS in nuclei | (Morelli et al., 2017; Tiago et al., 2021) |
|  | p.Y118H | |  | |  |  |  |  |  |  |  |  | (Nam et al., 2018) |
| HSPB4 | p.W9X | | ↓ | |  |  |  | ↓ |  |  | KO mouse |  | (Brady et al., 1997; Pras et al., 2000) |
|  | p.R12C/L | | ↓ | |  | ↑ |  | ↓ |  |  |  | impaired response to heat shock and oxidative stress | (Zhang et al., 2009; Khoshaman et al., 2015; Song et al., 2018) |
|  | p.R21C/W/L/Q | |  | | ↓ |  |  |  |  |  |  |  | (Graw et al., 2006; Laurie et al., 2013) |
|  | p.R49C | |  | |  | ↑ |  | ↓ autophagy | ↓ | ↑ | mouse | UPR pathway activation | (Mackay et al., 2003; Andley et al., 2008; Xi et al., 2008; Watson and Andley, 2010; Andley and Goldman, 2016) |
|  | p.R54C/P/L | | ↓ | |  | ↑ |  | ↓ / = | ↓ | ↑ | mouse |  | (Chang et al., 1996, 1999; Xia et al., 2006; Khoshaman et al., 2017; Ahsan et al., 2021) |
|  | p.R65Q | |  | |  |  |  |  |  |  |  |  |  |
|  | p.F71L | |  | |  |  |  | ↓ |  |  |  |  | (Bhagyalaxmi et al., 2009) |
|  | p.G98R | |  | |  | ↑ |  |  | ↓ |  |  | ER stress | (Singh et al., 2006) |
|  | p.R116C/H | |  | |  | ↑ |  |  |  |  |  |  | (Litt et al., 1998; Li et al., 2010; Pang et al., 2010; Zhang et al., 2011)(Andley et al., 2002) |
|  | p.116-118del | |  | |  | ↑ |  |  | ↓ |  | Y118D in mouse | UPR pathway activation | (Xia et al., 2006; Li et al., 2017; Jia et al., 2021) |
|  | p.117delR | |  | |  |  |  |  |  |  |  |  |  |
|  | p.R117H,Y118  del | |  | |  |  |  |  |  |  | Y118D in mouse |  | (Xia et al., 2006; Jia et al., 2021) |
|  | R119H | |  | |  |  |  |  |  |  |  |  |  |
|  | p.L139P | |  | |  | ↑ |  |  |  |  |  |  | (Liang et al., 2015) |
|  | p.Q147Rfs*48 | |  | |  |  |  |  |  |  |  |  |  |
|  | p.*174Qext*41; p.*174Sext*41 | |  | |  |  |  |  |  |  |  |  |  |
| HSPB5 | p.M1X | | ↓ | |  |  | ↓ |  | ↓ | ↑ | KO mouse |  | (Brady et al., 2001; Ma et al., 2019; Lu et al., 2021) |
|  | p.R11C/H | |  | |  | ↑ |  |  |  |  |  |  | (Raju and Abraham, 2013) |
|  | p.P20R/S | |  | |  | ↑ |  |  |  |  |  |  | (Li et al., 2008; Raju and Abraham, 2013) |
|  | p.S21Afs*24 | | ↓ | |  | ↑ |  | ↓ |  |  |  |  | (Del Bigio et al., 2011) |
|  | p.R56W | |  | |  |  |  |  |  |  |  |  |  |
|  | p.R69C | |  | |  | ↑ |  |  |  |  |  |  | (Ghahramani et al., 2020) |
|  | p.D109A/H/G | |  | |  | ↑ |  |  | ↓ |  |  |  | (Raju and Abraham, 2013; Brodehl et al., 2017; Fichna et al., 2017) |
|  | p.S115Pfs*14 | | ↓ | |  | ↑ |  | ↓ |  |  |  |  | (Forrest et al., 2011) |
|  | p.R120G | |  | |  | ↑ |  | ↓ | ↓ | ↑ | mouse | altered mitochondrial activity; ER stress and calcium homeostasis dysregulation | (Vicart et al., 1998; Chávez Zobel et al., 2003; Chen et al., 2005; Inagaki et al., 2006; Simon et al., 2007b; Tannous et al., 2008; Michiel et al., 2009; Clark et al., 2011; Pattison et al., 2011; Nivon et al., 2016; Brodehl et al., 2017) |
|  | p.R123W | |  | |  |  |  |  |  |  |  |  |  |
|  | p.D140N | |  | |  | ↑ |  |  |  |  |  |  | (Raju and Abraham, 2013) |
|  | p.K150Nfs*34 | |  | |  | ↑ |  | ↓ |  |  |  |  | (Hayes et al., 2008; Zhang et al., 2010) |
|  | p.Q151X | | ↓ | |  | ↑ |  | ↓ | ↓ |  |  |  | (Selcen and Engel, 2003; Simon et al., 2007a; Hayes et al., 2008; Bortolani et al., 2020) |
|  | p.P155Rfs*9 | | ↓ | |  | ↑ |  | ↓ | ↓ |  |  |  | (Selcen and Engel, 2003; Simon et al., 2007a; Hayes et al., 2008; Zhang et al., 2010) |
|  | p.G154S | |  | |  | ↑ |  | ↓ |  |  |  |  | (Reilich et al., 2010) |
|  | p.R157H | | ↓ | |  | ↓ |  | ↑ |  | ↑ |  |  | (Inagaki et al., 2006; Raju and Abraham, 2013; Nasiri et al., 2021) |
|  | p.A171T | |  | |  | ↑ |  |  | ↓ |  |  |  | (Raju and Abraham, 2013) |
|  | p.A172Pfs*14 | |  | |  |  |  |  |  |  |  |  |  |
|  | p.X176Wext*19 | |  | |  | ↑ |  |  |  |  |  |  | (van der Smagt et al., 2014) |
| HSPB6 | p.S10F | |  | |  | ↑ | = with HSPB1/HSPB5 | ↓ autophagy | ↓ |  | mouse | gender-related differences in mice models | (Liu et al., 2018b, 2018a; Shatov and Gusev, 2020) |
|  | p.P20L | | = | |  | ↑ | = with HSPB1/HSPB5 |  | = / ↓ upon I/R |  |  | ↑ phosphorylation rate | (Nicolaou et al., 2008; Shatov and Gusev, 2020) |
| HSPB8 | p.K141E/N/T/M | |  | | ↑ | ↑/ = | ↑/↓ with BAG3 | ↓ | ↓ | = | mouse / fly | mitochondrial abnormalities; altered redox system; altered RNA metabolism | (Irobi et al., 2004, 2012; Carra et al., 2005; Fontaine et al., 2006; Crippa et al., 2010; Shemetov and Gusev, 2011; Sanbe et al., 2013; Yang et al., 2017; Echaniz-Laguna et al., 2017a; Jo et al., 2017; Bouhy et al., 2018; Yu et al., 2019) |
|  | p.P90L | | = | |  | = | = with BAG3 |  | slight ↓ | = |  |  | (Echaniz-Laguna et al., 2017a) |
|  | p.N138T | | = | |  | = | = |  | slight ↓ | = |  |  | (Echaniz-Laguna et al., 2017a) |
|  | p.P173Sfs*43 | | ↓ | |  | ↑ (*in silico*) |  | ↓ |  |  |  |  | (Ghaoui et al., 2016; Al-Tahan et al., 2019; Inoue-Shibui et al., 2021) |
|  | p.Q170Gfs*45 | | ↓ | |  | ↑ (*in silico*) |  |  |  | ↑ |  | aggregates and rimmed vacuoles in skeletal muscle | (Echaniz-Laguna et al., 2017b; Inoue-Shibui et al., 2021) |
|  | p.T194Sfs*23 | |  | |  | ↑ (*in silico*) |  |  |  | ↑ |  | altered TIA-1 (SGs) distribution; aggregates and rimmed vacuoles in skeletal muscle | (Nicolau et al., 2020; Inoue-Shibui et al., 2021) |
|  | p.T176Wfs*38 | |  | |  | ↑ (*in silico*) |  |  |  |  |  |  | (Inoue-Shibui et al., 2021) |

**References**

Ackerley, S., James, P. A., Kalli, A., French, S., Davies, K. E., and Talbot, K. (2006). A mutation in the small heat-shock protein HSPB1 leading to distal hereditary motor neuronopathy disrupts neurofilament assembly and the axonal transport of specific cellular cargoes. *Hum. Mol. Genet.* 15, 347–54. doi:10.1093/hmg/ddi452.

Ahsan, S. M., Bakthisaran, R., Tangirala, R., and Rao, C. M. (2021). Nucleosomal association and altered interactome underlie the mechanism of cataract caused by the R54C mutation of αA-crystallin. *Biochim. Biophys. acta. Gen. Subj.* 1865, 129846. doi:10.1016/j.bbagen.2021.129846.

Al-Tahan, S., Weiss, L., Yu, H., Tang, S., Saporta, M., Vihola, A., et al. (2019). New family with HSPB8-associated autosomal dominant rimmed vacuolar myopathy. *Neurol. Genet.* 5, e349. doi:10.1212/NXG.0000000000000349.

Almeida-Souza, L., Goethals, S., de Winter, V., Dierick, I., Gallardo, R., Van Durme, J., et al. (2010). Increased monomerization of mutant HSPB1 leads to protein hyperactivity in Charcot-Marie-Tooth neuropathy. *J. Biol. Chem.* 285, 12778–86. doi:10.1074/jbc.M109.082644.

Amornvit, J., Yalvac, M. E., Chen, L., and Sahenk, Z. (2017). A novel p.T139M mutation in HSPB1 highlighting the phenotypic spectrum in a family. *Brain Behav.* 7, e00774. doi:10.1002/brb3.774.

Andley, U. P., and Goldman, J. W. (2016). Autophagy and UPR in alpha-crystallin mutant knock-in mouse models of hereditary cataracts. *Biochim. Biophys. Acta* 1860, 234–9. doi:10.1016/j.bbagen.2015.06.001.

Andley, U. P., Hamilton, P. D., and Ravi, N. (2008). Mechanism of insolubilization by a single-point mutation in alphaA-crystallin linked with hereditary human cataracts. *Biochemistry* 47, 9697–706. doi:10.1021/bi800594t.

Andley, U. P., Patel, H. C., and Xi, J.-H. (2002). The R116C mutation in alpha A-crystallin diminishes its protective ability against stress-induced lens epithelial cell apoptosis. *J. Biol. Chem.* 277, 10178–86. doi:10.1074/jbc.M109211200.

Benedetti, S., Previtali, S. C., Coviello, S., Scarlato, M., Cerri, F., Di Pierri, E., et al. (2010). Analyzing histopathological features of rare charcot-marie-tooth neuropathies to unravel their pathogenesis. *Arch. Neurol.* 67, 1498–505. doi:10.1001/archneurol.2010.303.

Berry, V., Francis, P., Reddy, M. A., Collyer, D., Vithana, E., MacKay, I., et al. (2001). Alpha-B crystallin gene (CRYAB) mutation causes dominant congenital posterior polar cataract in humans. *Am. J. Hum. Genet.* 69, 1141–5. doi:10.1086/324158.

Bhagyalaxmi, S. G., Srinivas, P., Barton, K. A., Kumar, K. R., Vidyavathi, M., Petrash, J. M., et al. (2009). A novel mutation (F71L) in alphaA-crystallin with defective chaperone-like function associated with age-related cataract. *Biochim. Biophys. Acta* 1792, 974–81. doi:10.1016/j.bbadis.2009.06.011.

Bortolani, S., Fattori, F., Monforte, M., Ricci, E., and Tasca, G. (2020). Peculiar muscle imaging findings in a patient with alphaB-crystallinopathy and axial myopathy. *J. Neurol. Sci.* 416, 116999. doi:10.1016/j.jns.2020.116999.

Bouhy, D., Juneja, M., Katona, I., Holmgren, A., Asselbergh, B., De Winter, V., et al. (2018). A knock-in/knock-out mouse model of HSPB8-associated distal hereditary motor neuropathy and myopathy reveals toxic gain-of-function of mutant Hspb8. *Acta Neuropathol* 135, 131–148. doi:10.1007/s00401-017-1756-0.

Brady, J. P., Garland, D., Duglas-Tabor, Y., Robison, W. G., Groome, A., and Wawrousek, E. F. (1997). Targeted disruption of the mouse alpha A-crystallin gene induces cataract and cytoplasmic inclusion bodies containing the small heat shock protein alpha B-crystallin. *Proc Natl Acad Sci U S A* 94, 884–889. doi:10.1073/pnas.94.3.884.

Brady, J. P., Garland, D. L., Green, D. E., Tamm, E. R., Giblin, F. J., and Wawrousek, E. F. (2001). AlphaB-crystallin in lens development and muscle integrity: a gene knockout approach. *Invest. Ophthalmol. Vis. Sci.* 42, 2924–34. Available at: http://www.ncbi.nlm.nih.gov/pubmed/11687538.

Brodehl, A., Gaertner-Rommel, A., Klauke, B., Grewe, S. A., Schirmer, I., Peterschröder, A., et al. (2017). The novel αB-crystallin (CRYAB) mutation p.D109G causes restrictive cardiomyopathy. *Hum. Mutat.* 38, 947–952. doi:10.1002/humu.23248.

Bugiardini, E., Rossor, A. M., Lynch, D. S., Swash, M., Pittman, A. M., Blake, J. C., et al. (2017). Homozygous mutation in HSPB1 causing distal vacuolar myopathy and motor neuropathy. *Neurol. Genet.* 3, e168. doi:10.1212/NXG.0000000000000168.

Capponi, S., Geroldi, A., Fossa, P., Grandis, M., Ciotti, P., Gulli, R., et al. (2011). HSPB1 and HSPB8 in inherited neuropathies: study of an Italian cohort of dHMN and CMT2 patients. *J. Peripher. Nerv. Syst.* 16, 287–94. doi:10.1111/j.1529-8027.2011.00361.x.

Capponi, S., Geuens, T., Geroldi, A., Origone, P., Verdiani, S., Cichero, E., et al. (2016a). Molecular Chaperones in the Pathogenesis of Amyotrophic Lateral Sclerosis: The Role of HSPB1. *Hum. Mutat.* 37, 1202–1208. doi:10.1002/humu.23062.

Capponi, S., Geuens, T., Geroldi, A., Origone, P., Verdiani, S., Cichero, E., et al. (2016b). Molecular Chaperones in the Pathogenesis of Amyotrophic Lateral Sclerosis: The Role of HSPB1. *Hum Mutat* 37, 1202–1208. doi:10.1002/humu.23062.

Carra, S., Sivilotti, M., Chávez Zobel, A. T., Lambert, H., and Landry, J. (2005). HspB8, a small heat shock protein mutated in human neuromuscular disorders, has in vivo chaperone activity in cultured cells. *Hum Mol Genet* 14, 1659–1669. doi:10.1093/hmg/ddi174.

Chalova, A. S., Sudnitsyna, M. V, Strelkov, S. V, and Gusev, N. B. (2014). Characterization of human small heat shock protein HspB1 that carries C-terminal domain mutations associated with hereditary motor neuron diseases. *Biochim. Biophys. Acta* 1844, 2116–26. doi:10.1016/j.bbapap.2014.09.005.

Chang, B., Hawes, N. L., Roderick, T. H., Smith, R. S., Heckenlively, J. R., Horwitz, J., et al. (1999). Identification of a missense mutation in the alphaA-crystallin gene of the lop18 mouse. *Mol. Vis.* 5, 21. Available at: http://www.ncbi.nlm.nih.gov/pubmed/10493778.

Chang, B., Hawes, N. L., Smith, R. S., Heckenlively, J. R., Davisson, M. T., and Roderick, T. H. (1996). Chromosomal localization of a new mouse lens opacity gene (lop18). *Genomics* 36, 171–3. doi:10.1006/geno.1996.0439.

Chávez Zobel, A. T., Loranger, A., Marceau, N., Thériault, J. R., Lambert, H., and Landry, J. (2003). Distinct chaperone mechanisms can delay the formation of aggresomes by the myopathy-causing R120G alphaB-crystallin mutant. *Hum. Mol. Genet.* 12, 1609–20. doi:10.1093/hmg/ddg173.

Chen, Q., Liu, J.-B., Horak, K. M., Zheng, H., Kumarapeli, A. R. K., Li, J., et al. (2005). Intrasarcoplasmic amyloidosis impairs proteolytic function of proteasomes in cardiomyocytes by compromising substrate uptake. *Circ. Res.* 97, 1018–26. doi:10.1161/01.RES.0000189262.92896.0b.

Chen, Q., Ma, J., Yan, M., Mothobi, M. E., Liu, Y., and Zheng, F. (2009). A novel mutation in CRYAB associated with autosomal dominant congenital nuclear cataract in a Chinese family. *Mol. Vis.* 15, 1359–65. Available at: http://www.ncbi.nlm.nih.gov/pubmed/19597569.

Clark, A. R., Naylor, C. E., Bagnéris, C., Keep, N. H., and Slingsby, C. (2011). Crystal structure of R120G disease mutant of human αB-crystallin domain dimer shows closure of a groove. *J Mol Biol* 408, 118–134. doi:10.1016/j.jmb.2011.02.020.

Clark, A. R., Vree Egberts, W., Kondrat, F. D. L., Hilton, G. R., Ray, N. J., Cole, A. R., et al. (2018). Terminal Regions Confer Plasticity to the Tetrameric Assembly of Human HspB2 and HspB3. *J. Mol. Biol.* 430, 3297–3310. doi:10.1016/j.jmb.2018.06.047.

Crippa, V., Sau, D., Rusmini, P., Boncoraglio, A., Onesto, E., Bolzoni, E., et al. (2010). The small heat shock protein B8 (HspB8) promotes autophagic removal of misfolded proteins involved in amyotrophic lateral sclerosis (ALS). *Hum Mol Genet* 19, 3440–3456. doi:10.1093/hmg/ddq257.

d’Ydewalle, C., Krishnan, J., Chiheb, D. M., Van Damme, P., Irobi, J., Kozikowski, A. P., et al. (2011). HDAC6 inhibitors reverse axonal loss in a mouse model of mutant HSPB1-induced Charcot-Marie-Tooth disease. *Nat Med* 17, 968–974. doi:10.1038/nm.2396.

Del Bigio, M. R., Chudley, A. E., Sarnat, H. B., Campbell, C., Goobie, S., Chodirker, B. N., et al. (2011). Infantile muscular dystrophy in Canadian aboriginals is an αB-crystallinopathy. *Ann Neurol* 69, 866–871. doi:10.1002/ana.22331.

Devi, R. R., Yao, W., Vijayalakshmi, P., Sergeev, Y. V, Sundaresan, P., and Hejtmancik, J. F. (2008). Crystallin gene mutations in Indian families with inherited pediatric cataract. *Mol. Vis.* 14, 1157–70. Available at: http://www.ncbi.nlm.nih.gov/pubmed/18587492.

Dierick, I., Baets, J., Irobi, J., Jacobs, A., De Vriendt, E., Deconinck, T., et al. (2008). Relative contribution of mutations in genes for autosomal dominant distal hereditary motor neuropathies: a genotype-phenotype correlation study. *Brain* 131, 1217–27. doi:10.1093/brain/awn029.

DiVincenzo, C., Elzinga, C. D., Medeiros, A. C., Karbassi, I., Jones, J. R., Evans, M. C., et al. (2014). The allelic spectrum of Charcot-Marie-Tooth disease in over 17,000 individuals with neuropathy. *Mol. Genet. genomic Med.* 2, 522–9. doi:10.1002/mgg3.106.

Echaniz-Laguna, A., Geuens, T., Petiot, P., Péréon, Y., Adriaenssens, E., Haidar, M., et al. (2017a). Axonal Neuropathies due to Mutations in Small Heat Shock Proteins: Clinical, Genetic, and Functional Insights into Novel Mutations. *Hum. Mutat.* 38, 556–568. doi:10.1002/humu.23189.

Echaniz-Laguna, A., Lornage, X., Lannes, B., Schneider, R., Bierry, G., Dondaine, N., et al. (2017b). HSPB8 haploinsufficiency causes dominant adult-onset axial and distal myopathy. *Acta Neuropathol* 134, 163–165. doi:10.1007/s00401-017-1724-8.

Evgrafov, O. V, Mersiyanova, I., Irobi, J., Van Den Bosch, L., Dierick, I., Leung, C. L., et al. (2004). Mutant small heat-shock protein 27 causes axonal Charcot-Marie-Tooth disease and distal hereditary motor neuropathy. *Nat. Genet.* 36, 602–6. doi:10.1038/ng1354.

Fichna, J. P., Potulska-Chromik, A., Miszta, P., Redowicz, M. J., Kaminska, A. M., Zekanowski, C., et al. (2017). A novel dominant D109A CRYAB mutation in a family with myofibrillar myopathy affects αB-crystallin structure. *BBA Clin.* 7, 1–7. doi:10.1016/j.bbacli.2016.11.004.

Fontaine, J. M., Sun, X., Hoppe, A. D., Simon, S., Vicart, P., Welsh, M. J., et al. (2006). Abnormal small heat shock protein interactions involving neuropathy-associated HSP22 (HSPB8) mutants. *FASEB J* 20, 2168–2170. doi:10.1096/fj.06-5911fje.

Forrest, K. M., Al-Sarraj, S., Sewry, C., Buk, S., Tan, S. V, Pitt, M., et al. (2011). Infantile onset myofibrillar myopathy due to recessive CRYAB mutations. *Neuromuscul Disord* 21, 37–40. doi:10.1016/j.nmd.2010.11.003.

Fortunato, F., Neri, M., Geroldi, A., Bellone, E., De Grandis, D., Ferlini, A., et al. (2017). A CMT2 family carrying the P7R mutation in the N- terminal region of the HSPB1 gene. *Clin. Neurol. Neurosurg.* 163, 15–17. doi:10.1016/j.clineuro.2017.09.012.

Frasquet, M., Rojas-García, R., Argente-Escrig, H., Vázquez-Costa, J. F., Muelas, N., Vílchez, J. J., et al. (2021). Distal hereditary motor neuropathies: Mutation spectrum and genotype-phenotype correlation. *Eur. J. Neurol.* 28, 1334–1343. doi:10.1111/ene.14700.

Ghahramani, M., Yousefi, R., Krivandin, A., Muranov, K., Kurganov, B., and Moosavi-Movahedi, A. A. (2020). Structural and functional characterization of D109H and R69C mutant versions of human αB-crystallin: The biochemical pathomechanism underlying cataract and myopathy development. *Int. J. Biol. Macromol.* 146, 1142–1160. doi:10.1016/j.ijbiomac.2019.09.239.

Ghaoui, R., Palmio, J., Brewer, J., Lek, M., Needham, M., Evilä, A., et al. (2016). Mutations in HSPB8 causing a new phenotype of distal myopathy and motor neuropathy. *Neurology* 86, 391–398. doi:10.1212/WNL.0000000000002324.

Graw, J., Klopp, N., Illig, T., Preising, M. N., and Lorenz, B. (2006). Congenital cataract and macular hypoplasia in humans associated with a de novo mutation in CRYAA and compound heterozygous mutations in P. *Graefes Arch. Clin. Exp. Ophthalmol.* 244, 912–9. doi:10.1007/s00417-005-0234-x.

Haidar, M., Asselbergh, B., Adriaenssens, E., De Winter, V., Timmermans, J.-P. P., Auer-Grumbach, M., et al. (2019). Neuropathy-causing mutations in HSPB1 impair autophagy by disturbing the formation of SQSTM1/p62 bodies. *Autophagy* 15, 1051–1068. doi:10.1080/15548627.2019.1569930.

Hansen, L., Yao, W., Eiberg, H., Kjaer, K. W., Baggesen, K., Hejtmancik, J. F., et al. (2007). Genetic heterogeneity in microcornea-cataract: five novel mutations in CRYAA, CRYGD, and GJA8. *Invest. Ophthalmol. Vis. Sci.* 48, 3937–44. doi:10.1167/iovs.07-0013.

Hayes, V. H., Devlin, G., and Quinlan, R. A. (2008). Truncation of alphaB-crystallin by the myopathy-causing Q151X mutation significantly destabilizes the protein leading to aggregate formation in transfected cells. *J. Biol. Chem.* 283, 10500–12. doi:10.1074/jbc.M706453200.

Holmgren, A., Bouhy, D., De Winter, V., Asselbergh, B., Timmermans, J.-P., Irobi, J., et al. (2013). Charcot-Marie-Tooth causing HSPB1 mutations increase Cdk5-mediated phosphorylation of neurofilaments. *Acta Neuropathol.* 126, 93–108. doi:10.1007/s00401-013-1133-6.

Houlden, H., Laura, M., Wavrant-De Vrièze, F., Blake, J., Wood, N., and Reilly, M. M. (2008). Mutations in the HSP27 (HSPB1) gene cause dominant, recessive, and sporadic distal HMN/CMT type 2. *Neurology* 71, 1660–1668. doi:10.1212/01.wnl.0000319696.14225.67.

Ikeda, Y., Abe, A., Ishida, C., Takahashi, K., Hayasaka, K., and Yamada, M. (2009). A clinical phenotype of distal hereditary motor neuronopathy type II with a novel HSPB1 mutation. *J. Neurol. Sci.* 277, 9–12. doi:10.1016/j.jns.2008.09.031.

Inagaki, N., Hayashi, T., Arimura, T., Koga, Y., Takahashi, M., Shibata, H., et al. (2006). Alpha B-crystallin mutation in dilated cardiomyopathy. *Biochem. Biophys. Res. Commun.* 342, 379–86. doi:10.1016/j.bbrc.2006.01.154.

Inoue-Shibui, A., Niihori, T., Kobayashi, M., Suzuki, N., Izumi, R., Warita, H., et al. (2021). A novel deletion in the C-terminal region of HSPB8 in a family with rimmed vacuolar myopathy. *J. Hum. Genet.* 66, 965–972. doi:10.1038/s10038-021-00916-y.

Irobi, J., Holmgren, A., De Winter, V., Asselbergh, B., Gettemans, J., Adriaensen, D., et al. (2012). Mutant HSPB8 causes protein aggregates and a reduced mitochondrial membrane potential in dermal fibroblasts from distal hereditary motor neuropathy patients. *Neuromuscul Disord* 22, 699–711. doi:10.1016/j.nmd.2012.04.005.

Irobi, J., Van Impe, K., Seeman, P., Jordanova, A., Dierick, I., Verpoorten, N., et al. (2004). Hot-spot residue in small heat-shock protein 22 causes distal motor neuropathy. *Nat Genet* 36, 597–601. doi:10.1038/ng1328.

Ismailov, S. M., Fedotov, V. P., Dadali, E. L., Polyakov, A. V, Van Broeckhoven, C., Ivanov, V. I., et al. (2001). A new locus for autosomal dominant Charcot-Marie-Tooth disease type 2 (CMT2F) maps to chromosome 7q11-q21. *Eur. J. Hum. Genet.* 9, 646–50. doi:10.1038/sj.ejhg.5200686.

James, P. A., Rankin, J., and Talbot, K. (2008). Asymmetrical late onset motor neuropathy associated with a novel mutation in the small heat shock protein HSPB1 (HSP27). *J. Neurol. Neurosurg. Psychiatry* 79, 461–3. doi:10.1136/jnnp.2007.125179.

Javadiyan, S., Craig, J. E., Souzeau, E., Sharma, S., Lower, K. M., Mackey, D. A., et al. (2017). High-Throughput Genetic Screening of 51 Pediatric Cataract Genes Identifies Causative Mutations in Inherited Pediatric Cataract in South Eastern Australia. *G3 (Bethesda).* 7, 3257–3268. doi:10.1534/g3.117.300109.

Jia, Z.-K., Fu, C.-X., Wang, A.-L., Yao, K., and Chen, X.-J. (2021). Cataract-causing allele in CRYAA (Y118D) proceeds through endoplasmic reticulum stress in mouse model. *Zool. Res.* 42, 300–309. doi:10.24272/j.issn.2095-8137.2020.354.

Jiao, X., Khan, S. Y., Irum, B., Khan, A. O., Wang, Q., Kabir, F., et al. (2015). Missense Mutations in CRYAB Are Liable for Recessive Congenital Cataracts. *PLoS One* 10, e0137973. doi:10.1371/journal.pone.0137973.

Jo, H. S., Kim, D. W., Shin, M. J., Cho, S. B., Park, J. H., Lee, C. H., et al. (2017). Tat-HSP22 inhibits oxidative stress-induced hippocampal neuronal cell death by regulation of the mitochondrial pathway. *Mol Brain* 10, 1. doi:10.1186/s13041-016-0281-8.

Kalmar, B., Innes, A., Wanisch, K., Kolaszynska, A. K., Pandraud, A., Kelly, G., et al. (2017). Mitochondrial deficits and abnormal mitochondrial retrograde axonal transport play a role in the pathogenesis of mutant Hsp27-induced Charcot Marie Tooth Disease. *Hum Mol Genet* 26, 3313–3326. doi:10.1093/hmg/ddx216.

Kang, K.-H., Han, J. E., Hong, Y. Bin, Nam, S. H., Choi, B.-O., and Koh, H. (2020). Human HSPB1 mutation recapitulates features of distal hereditary motor neuropathy (dHMN) in Drosophila. *Biochem. Biophys. Res. Commun.* 521, 220–226. doi:10.1016/j.bbrc.2019.10.110.

Khan, A. O., Abu Safieh, L., Alkuraya, F. S., and Alkurarya, F. S. (2010). Later retinal degeneration following childhood surgical aphakia in a family with recessive CRYAB mutation (p.R56W). *Ophthalmic Genet.* 31, 30–6. doi:10.3109/13816810903452047.

Khan, A. O., Aldahmesh, M. A., and Meyer, B. (2007). Recessive congenital total cataract with microcornea and heterozygote carrier signs caused by a novel missense CRYAA mutation (R54C). *Am. J. Ophthalmol.* 144, 949–952. doi:10.1016/j.ajo.2007.08.005.

Khoshaman, K., Yousefi, R., Tamaddon, A. M., Abolmaali, S. S., Oryan, A., Moosavi-Movahedi, A. A., et al. (2017). The impact of different mutations at Arg54 on structure, chaperone-like activity and oligomerization state of human αA-crystallin: The pathomechanism underlying congenital cataract-causing mutations R54L, R54P and R54C. *Biochim. Biophys. acta. Proteins proteomics* 1865, 604–618. doi:10.1016/j.bbapap.2017.02.003.

Khoshaman, K., Yousefi, R., Tamaddon, A. M., Saso, L., and Moosavi-Movahedi, A. A. (2015). The impact of Hydrogen peroxide on structure, stability and functional properties of Human R12C mutant αA-crystallin: The imperative insights into pathomechanism of the associated congenital cataract incidence. *Free Radic. Biol. Med.* 89, 819–30. doi:10.1016/j.freeradbiomed.2015.09.013.

Kijima, K., Numakura, C., Goto, T., Takahashi, T., Otagiri, T., Umetsu, K., et al. (2005). Small heat shock protein 27 mutation in a Japanese patient with distal hereditary motor neuropathy. *J. Hum. Genet.* 50, 473–476. doi:10.1007/s10038-005-0280-6.

Kolb, S. J., Snyder, P. J., Poi, E. J., Renard, E. A., Bartlett, A., Gu, S., et al. (2010). Mutant small heat shock protein B3 causes motor neuropathy: utility of a candidate gene approach. *Neurology* 74, 502–506. doi:10.1212/WNL.0b013e3181cef84a.

Kong, X. D., Liu, N., Shi, H. R., Dong, J. M., Zhao, Z. H., Liu, J., et al. (2015). A novel 3-base pair deletion of the CRYAA gene identified in a large Chinese pedigree featuring autosomal dominant congenital perinuclear cataract. *Genet. Mol. Res.* 14, 426–32. doi:10.4238/2015.January.23.16.

Laurie, K. J., Dave, A., Straga, T., Souzeau, E., Chataway, T., Sykes, M. J., et al. (2013). Identification of a novel oligomerization disrupting mutation in CRYΑA associated with congenital cataract in a South Australian family. *Hum. Mutat.* 34, 435–8. doi:10.1002/humu.22260.

Lee, J., Jung, S.-C., Joo, J., Choi, Y.-R., Moon, H. W., Kwak, G., et al. (2015). Overexpression of mutant HSP27 causes axonal neuropathy in mice. *J. Biomed. Sci.* 22, 43. doi:10.1186/s12929-015-0154-y.

Lewis-Smith, D. J., Duff, J., Pyle, A., Griffin, H., Polvikoski, T., Birchall, D., et al. (2016). Novel HSPB1 mutation causes both motor neuronopathy and distal myopathy. *Neurol. Genet.* 2, e110. doi:10.1212/NXG.0000000000000110.

Li, F.-F., Yang, M., Ma, X., Zhang, Q., Zhang, M., Wang, S.-Z., et al. (2010). Autosomal dominant congenital nuclear cataracts caused by a CRYAA gene mutation. *Curr. Eye Res.* 35, 492–8. doi:10.3109/02713681003624901.

Li, H., Li, C., Lu, Q., Su, T., Ke, T., Li, D. W.-C., et al. (2008). Cataract mutation P20S of alphaB-crystallin impairs chaperone activity of alphaA-crystallin and induces apoptosis of human lens epithelial cells. *Biochim. Biophys. Acta* 1782, 303–9. doi:10.1016/j.bbadis.2008.01.011.

Li, L., Fan, D.-B., Zhao, Y.-T., Li, Y., Kong, D.-Q., Cai, F.-F., et al. (2017). Two novel mutations identified in ADCC families impair crystallin protein distribution and induce apoptosis in human lens epithelial cells. *Sci. Rep.* 7, 17848. doi:10.1038/s41598-017-18222-z.

Liang, C., Liang, H., Yang, Y., Ping, L., and Jie, Q. (2015). Mutation analysis of two families with inherited congenital cataracts. *Mol. Med. Rep.* 12, 3469–3475. doi:10.3892/mmr.2015.3819.

Lin, K.-P., Soong, B.-W., Yang, C.-C., Huang, L.-W., Chang, M.-H., Lee, I.-H., et al. (2011). The mutational spectrum in a cohort of Charcot-Marie-Tooth disease type 2 among the Han Chinese in Taiwan. *PLoS One* 6, e29393. doi:10.1371/journal.pone.0029393.

Litt, M., Kramer, P., LaMorticella, D. M., Murphey, W., Lovrien, E. W., and Weleber, R. G. (1998). Autosomal dominant congenital cataract associated with a missense mutation in the human alpha crystallin gene CRYAA. *Hum. Mol. Genet.* 7, 471–4. doi:10.1093/hmg/7.3.471.

Liu, G.-S., Gardner, G., Adly, G., Jiang, M., Cai, W.-F., Lam, C. K., et al. (2018a). A novel human S10F-Hsp20 mutation induces lethal peripartum cardiomyopathy. *J. Cell. Mol. Med.* doi:10.1111/jcmm.13665.

Liu, G.-S., Zhu, H., Cai, W.-F., Wang, X., Jiang, M., Essandoh, K., et al. (2018b). Regulation of BECN1-mediated autophagy by HSPB6: Insights from a human HSPB6S10F mutant. *Autophagy* 14, 80–97. doi:10.1080/15548627.2017.1392420.

Liu, M., Ke, T., Wang, Z., Yang, Q., Chang, W., Jiang, F., et al. (2006a). Identification of a CRYAB mutation associated with autosomal dominant posterior polar cataract in a Chinese family. *Invest. Ophthalmol. Vis. Sci.* 47, 3461–6. doi:10.1167/iovs.05-1438.

Liu, Y., Zhang, X., Luo, L., Wu, M., Zeng, R., Cheng, G., et al. (2006b). A novel alphaB-crystallin mutation associated with autosomal dominant congenital lamellar cataract. *Invest Ophthalmol Vis Sci* 47, 1069–1075. doi:10.1167/iovs.05-1004.

Lorefice, L., Murru, M. R., Coghe, G., Fenu, G., Corongiu, D., Frau, J., et al. (2017). Charcot-Marie-Tooth disease: genetic subtypes in the Sardinian population. *Neurol. Sci.* 38, 1019–1025. doi:10.1007/s10072-017-2905-x.

Lu, X.-G., Yu, U., Han, C.-X., Mai, J.-H., Liao, J.-X., and Hou, Y.-Q. (2021). c.3G>A mutation in the CRYAB gene that causes fatal infantile hypertonic myofibrillar myopathy in the Chinese population. *J. Integr. Neurosci.* 20, 143–151. doi:10.31083/j.jin.2021.01.267.

Luigetti, M., Fabrizi, G. M., Bisogni, G., Romano, A., Taioli, F., Ferrarini, M., et al. (2016). Charcot-Marie-Tooth type 2 and distal hereditary motor neuropathy: Clinical, neurophysiological and genetic findings from a single-centre experience. *Clin. Neurol. Neurosurg.* 144, 67–71. doi:10.1016/j.clineuro.2016.03.007.

Luigetti, M., Fabrizi, G. M., Madia, F., Ferrarini, M., Conte, A., Del Grande, A., et al. (2010). A novel HSPB1 mutation in an Italian patient with CMT2/dHMN phenotype. *J. Neurol. Sci.* 298, 114–7. doi:10.1016/j.jns.2010.09.008.

Ma, K., Luo, D., Tian, T., Li, N., He, X., Rao, C., et al. (2019). A novel homozygous initiation codon variant associated with infantile alpha-Bcrystallinopathy in a Chinese family. *Mol. Genet. genomic Med.* 7, e825. doi:10.1002/mgg3.825.

Mackay, D. S., Andley, U. P., and Shiels, A. (2003). Cell death triggered by a novel mutation in the alphaA-crystallin gene underlies autosomal dominant cataract linked to chromosome 21q. *Eur. J. Hum. Genet.* 11, 784–93. doi:10.1038/sj.ejhg.5201046.

Maeda, K., Idehara, R., Hashiguchi, A., and Takashima, H. (2014). A family with distal hereditary motor neuropathy and a K141Q mutation of small heat shock protein HSPB1. *Intern. Med.* 53, 1655–8. doi:10.2169/internalmedicine.53.2843.

Mandich, P., Grandis, M., Varese, A., Geroldi, A., Acquaviva, M., Ciotti, P., et al. (2010). Severe neuropathy after diphtheria-tetanus-pertussis vaccination in a child carrying a novel frame-shift mutation in the small heat-shock protein 27 gene. *J. Child Neurol.* 25, 107–9. doi:10.1177/0883073809334387.

Marakhonov, A. V, Voskresenskaya, A. A., Ballesta, M. J., Konovalov, F. A., Vasilyeva, T. A., Blanco-Kelly, F., et al. (2020). Expanding the phenotype of CRYAA nucleotide variants to a complex presentation of anterior segment dysgenesis. *Orphanet J. Rare Dis.* 15, 207. doi:10.1186/s13023-020-01484-8.

Marcos, A. T., Amorós, D., Muñoz-Cabello, B., Galán, F., Rivas Infante, E., Alcaraz-Mas, L., et al. (2020). A novel dominant mutation in CRYAB gene leading to a severe phenotype with childhood onset. *Mol. Genet. genomic Med.* 8, e1290. doi:10.1002/mgg3.1290.

Maron, B. J., Rowin, E. J., Arkun, K., Rastegar, H., Larson, A. M., Maron, M. S., et al. (2020). Adult Monozygotic Twins With Hypertrophic Cardiomyopathy and Identical Disease Expression and Clinical Course. *Am. J. Cardiol.* 127, 135–138. doi:10.1016/j.amjcard.2020.04.020.

Michiel, M., Skouri-Panet, F., Duprat, E., Simon, S., Férard, C., Tardieu, A., et al. (2009). Abnormal assemblies and subunit exchange of alphaB-crystallin R120 mutants could be associated with destabilization of the dimeric substructure. *Biochemistry* 48, 442–53. doi:10.1021/bi8014967.

Morelli, F. F., Verbeek, D. S., Bertacchini, J., Vinet, J., Mediani, L., Marmiroli, S., et al. (2017). Aberrant Compartment Formation by HSPB2 Mislocalizes Lamin A and Compromises Nuclear Integrity and Function. *Cell Rep.* 20, 2100–2115. doi:10.1016/j.celrep.2017.08.018.

Muranova, L. K., Weeks, S. D., Strelkov, S. V, and Gusev, N. B. (2015). Characterization of Mutants of Human Small Heat Shock Protein HspB1 Carrying Replacements in the N-Terminal Domain and Associated with Hereditary Motor Neuron Diseases. *PLoS One* 10, e0126248. doi:10.1371/journal.pone.0126248.

Nakhro, K., Park, J. M., Kim, Y. J., Yoon, B. R., Yoo, J. H., Koo, H., et al. (2013). A novel Lys141Thr mutation in small heat shock protein 22 (HSPB8) gene in Charcot-Marie-Tooth disease type 2L. *Neuromuscul Disord* 23, 656–663. doi:10.1016/j.nmd.2013.05.009.

Nam, D. E., Nam, S. H., Lee, A. J., Hong, Y. B., Choi, B. O., and Chung, K. W. (2018). Small heat shock protein B3 (HSPB3) mutation in an axonal Charcot-Marie-Tooth disease family. *J Peripher Nerv Syst* 23, 60–66. doi:10.1111/jns.12249.

Nasiri, P., Ghahramani, M., Tavaf, Z., Niazi, A., Moosavi-Movahedi, A. A., Kurganov, B. I., et al. (2021). The biochemical association between R157H mutation in human αB-crystallin and development of cardiomyopathy: Structural and functional analyses of the mutant protein. *Biochimie* 190, 36–49. doi:10.1016/j.biochi.2021.06.019.

Nefedova, V. V, Datskevich, P. N., Sudnitsyna, M. V, Strelkov, S. V, and Gusev, N. B. (2013a). Physico-chemical properties of R140G and K141Q mutants of human small heat shock protein HspB1 associated with hereditary peripheral neuropathies. *Biochimie* 95, 1582–92. doi:10.1016/j.biochi.2013.04.014.

Nefedova, V. V, Sudnitsyna, M. V, and Gusev, N. B. (2017). Interaction of small heat shock proteins with light component of neurofilaments (NFL). *Cell Stress Chaperones* 22, 467–479. doi:10.1007/s12192-016-0757-6.

Nefedova, V. V, Sudnitsyna, M. V, Strelkov, S. V, and Gusev, N. B. (2013b). Structure and properties of G84R and L99M mutants of human small heat shock protein HspB1 correlating with motor neuropathy. *Arch. Biochem. Biophys.* 538, 16–24. doi:10.1016/j.abb.2013.07.028.

Nicolaou, P., Knöll, R., Haghighi, K., Fan, G. C., Dorn, G. W., Hasenfub, G., et al. (2008). Human mutation in the anti-apoptotic heat shock protein 20 abrogates its cardioprotective effects. *J Biol Chem* 283, 33465–33471. doi:10.1074/jbc.M802307200.

Nicolau, S., Liewluck, T., Elliott, J. L., Engel, A. G., and Milone, M. (2020). A novel heterozygous mutation in the C-terminal region of HSPB8 leads to limb-girdle rimmed vacuolar myopathy. *Neuromuscul Disord*. doi:10.1016/j.nmd.2020.02.005.

Nivon, M., Fort, L., Muller, P., Richet, E., Simon, S., Guey, B., et al. (2016). NFκB is a central regulator of protein quality control in response to protein aggregation stresses via autophagy modulation. *Mol. Biol. Cell* 27, 1712–27. doi:10.1091/mbc.E15-12-0835.

Pang, M., Su, J.-T., Feng, S., Tang, Z.-W., Gu, F., Zhang, M., et al. (2010). Effects of congenital cataract mutation R116H on alphaA-crystallin structure, function and stability. *Biochim. Biophys. Acta* 1804, 948–56. doi:10.1016/j.bbapap.2010.01.001.

Patel, R., Zenith, R. K., Chandra, A., and Ali, A. (2017). Novel Mutations in the Crystallin Gene in Age-Related Cataract Patients from a North Indian Population. *Mol. Syndromol.* 8, 179–186. doi:10.1159/000471992.

Pattison, J. S., Osinska, H., and Robbins, J. (2011). Atg7 induces basal autophagy and rescues autophagic deficiency in CryABR120G cardiomyocytes. *Circ. Res.* 109, 151–60. doi:10.1161/CIRCRESAHA.110.237339.

Potulska-Chromik, A., Jędrzejowska, M., Gos, M., Rosiak, E., Kierdaszuk, B., Maruszak, A., et al. (2021). Pathogenic Mutations and Putative Phenotype-Affecting Variants in Polish Myofibrillar Myopathy Patients. *J. Clin. Med.* 10. doi:10.3390/jcm10050914.

Pras, E., Frydman, M., Levy-Nissenbaum, E., Bakhan, T., Raz, J., Assia, E. I., et al. (2000). A nonsense mutation (W9X) in CRYAA causes autosomal recessive cataract in an inbred Jewish Persian family. *Invest Ophthalmol Vis Sci* 41, 3511–3515. Available at: https://www.ncbi.nlm.nih.gov/pubmed/11006246.

Raju, I., and Abraham, E. C. (2013). Mutants of human αB-crystallin cause enhanced protein aggregation and apoptosis in mammalian cells: influence of co-expression of HspB1. *Biochem. Biophys. Res. Commun.* 430, 107–12. doi:10.1016/j.bbrc.2012.11.051.

Reilich, P., Schoser, B., Schramm, N., Krause, S., Schessl, J., Kress, W., et al. (2010). The p.G154S mutation of the alpha-B crystallin gene (CRYAB) causes late-onset distal myopathy. *Neuromuscul. Disord.* 20, 255–9. doi:10.1016/j.nmd.2010.01.012.

Rossor, A. M., Davidson, G. L., Blake, J., Polke, J. M., Murphy, S. M., Houlden, H., et al. (2012). A novel p.Gln175X [corrected] premature stop mutation in the C-terminal end of HSP27 is a cause of CMT2. *J. Peripher. Nerv. Syst.* 17, 201–5. doi:10.1111/j.1529-8027.2012.00400.x.

Rossor, A. M., Morrow, J. M., Polke, J. M., Murphy, S. M., Houlden, H., INC-RDCRC, et al. (2017). Pilot phenotype and natural history study of hereditary neuropathies caused by mutations in the HSPB1 gene. *Neuromuscul. Disord.* 27, 50–56. doi:10.1016/j.nmd.2016.10.001.

Sacconi, S., Féasson, L., Antoine, J. C., Pécheux, C., Bernard, R., Cobo, A. M., et al. (2012). A novel CRYAB mutation resulting in multisystemic disease. *Neuromuscul Disord* 22, 66–72. doi:10.1016/j.nmd.2011.07.004.

Safieh, L. A., Khan, A. O., and Alkuraya, F. S. (2009). Identification of a novel CRYAB mutation associated with autosomal recessive juvenile cataract in a Saudi family. *Mol. Vis.* 15, 980–4. Available at: http://www.ncbi.nlm.nih.gov/pubmed/19461931.

Sanbe, A., Marunouchi, T., Abe, T., Tezuka, Y., Okada, M., Aoki, S., et al. (2013). Phenotype of cardiomyopathy in cardiac-specific heat shock protein B8 K141N transgenic mouse. *J Biol Chem* 288, 8910–8921. doi:10.1074/jbc.M112.368324.

Santhiya, S. T., Soker, T., Klopp, N., Illig, T., Prakash, M. V. S., Selvaraj, B., et al. (2006). Identification of a novel, putative cataract-causing allele in CRYAA (G98R) in an Indian family. *Mol. Vis.* 12, 768–73. Available at: http://www.ncbi.nlm.nih.gov/pubmed/16862070.

Scarlato, M., Viganò, F., Carrera, P., Previtali, S. C., and Bolino, A. (2015). A novel heat shock protein 27 homozygous mutation: widening of the continuum between MND/dHMN/CMT2. *J. Peripher. Nerv. Syst.* 20, 419–21. doi:10.1111/jns.12139.

Selcen, D., and Engel, A. G. (2003). Myofibrillar myopathy caused by novel dominant negative alpha B-crystallin mutations. *Ann. Neurol.* 54, 804–10. doi:10.1002/ana.10767.

Shatov, V. M., and Gusev, N. B. (2020). Physico-chemical properties of two point mutants of small heat shock protein HspB6 (Hsp20) with abrogated cardioprotection. *Biochimie* 174, 126–135. doi:10.1016/j.biochi.2020.04.021.

Shemetov, A. A., and Gusev, N. B. (2011). Biochemical characterization of small heat shock protein HspB8 (Hsp22)-Bag3 interaction. *Arch. Biochem. Biophys.* 513, 1–9. doi:10.1016/j.abb.2011.06.014.

Simon, S., Fontaine, J.-M., Martin, J. L., Sun, X., Hoppe, A. D., Welsh, M. J., et al. (2007a). Myopathy-associated alphaB-crystallin mutants: abnormal phosphorylation, intracellular location, and interactions with other small heat shock proteins. *J. Biol. Chem.* 282, 34276–87. doi:10.1074/jbc.M703267200.

Simon, S., Michiel, M., Skouri-Panet, F., Lechaire, J. P., Vicart, P., and Tardieu, A. (2007b). Residue R120 is essential for the quaternary structure and functional integrity of human alphaB-crystallin. *Biochemistry* 46, 9605–14. doi:10.1021/bi7003125.

Singh, D., Raman, B., Ramakrishna, T., and Rao, C. M. (2006). The cataract-causing mutation G98R in human alphaA-crystallin leads to folding defects and loss of chaperone activity. *Mol. Vis.* 12, 1372–9. Available at: http://www.ncbi.nlm.nih.gov/pubmed/17149363.

Solla, P., Vannelli, A., Bolino, A., Marrosu, G., Coviello, S., Murru, M. R., et al. (2010). Heat shock protein 27 R127W mutation: evidence of a continuum between axonal Charcot-Marie-Tooth and distal hereditary motor neuropathy. *J. Neurol. Neurosurg. Psychiatry* 81, 958–62. doi:10.1136/jnnp.2009.181636.

Song, Z., Si, N., and Xiao, W. (2018). A novel mutation in the CRYAA gene associated with congenital cataract and microphthalmia in a Chinese family. *BMC Med. Genet.* 19, 190. doi:10.1186/s12881-018-0695-5.

Srivastava, A. K., Renusch, S. R., Naiman, N. E., Gu, S., Sneh, A., Arnold, W. D., et al. (2012). Mutant HSPB1 overexpression in neurons is sufficient to cause age-related motor neuronopathy in mice. *Neurobiol. Dis.* 47, 163–73. doi:10.1016/j.nbd.2012.03.035.

Su, D., Guo, Y., Li, Q., Guan, L., Zhu, S., and Ma, X. (2012). A novel mutation in CRYAA is associated with autosomal dominant suture cataracts in a Chinese family. *Mol. Vis.* 18, 3057–63. Available at: http://www.ncbi.nlm.nih.gov/pubmed/23288997.

Sun, W., Xiao, X., Li, S., Guo, X., and Zhang, Q. (2011). Mutation analysis of 12 genes in Chinese families with congenital cataracts. *Mol. Vis.* 17, 2197–206. Available at: http://www.ncbi.nlm.nih.gov/pubmed/21866213.

Tanabe, H., Higuchi, Y., Yuan, J.-H., Hashiguchi, A., Yoshimura, A., Ishihara, S., et al. (2018). Clinical and genetic features of Charcot-Marie-Tooth disease 2F and hereditary motor neuropathy 2B in Japan. *J. Peripher. Nerv. Syst.* 23, 40–48. doi:10.1111/jns.12252.

Tang, B., Liu, X., Zhao, G., Luo, W., Xia, K., Pan, Q., et al. (2005a). Mutation analysis of the small heat shock protein 27 gene in chinese patients with Charcot-Marie-Tooth disease. *Arch. Neurol.* 62, 1201–7. doi:10.1001/archneur.62.8.1201.

Tang, B., Zhao, G., Luo, W., Xia, K., Cai, F., Pan, Q., et al. (2005b). Small heat-shock protein 22 mutated in autosomal dominant Charcot-Marie-Tooth disease type 2L. *Hum. Genet.* 116, 222–4. doi:10.1007/s00439-004-1218-3.

Tannous, P., Zhu, H., Johnstone, J. L., Shelton, J. M., Rajasekaran, N. S., Benjamin, I. J., et al. (2008). Autophagy is an adaptive response in desmin-related cardiomyopathy. *Proc. Natl. Acad. Sci. U. S. A.* 105, 9745–50. doi:10.1073/pnas.0706802105.

Tiago, T., Hummel, B., Morelli, F. F., Basile, V., Vinet, J., Galli, V., et al. (2021). Small heat-shock protein HSPB3 promotes myogenesis by regulating the lamin B receptor. *Cell Death Dis.* 12, 452. doi:10.1038/s41419-021-03737-1.

van der Smagt, J. J., Vink, A., Kirkels, J. H., Nelen, M., ter Heide, H., Molenschot, M. M. C., et al. (2014). Congenital posterior pole cataract and adult onset dilating cardiomyopathy: expanding the phenotype of αB-crystallinopathies. *Clin. Genet.* 85, 381–5. doi:10.1111/cge.12169.

Vicart, P., Caron, A., Guicheney, P., Li, Z., Prévost, M. C., Faure, A., et al. (1998). A missense mutation in the alphaB-crystallin chaperone gene causes a desmin-related myopathy. *Nat. Genet.* 20, 92–5. doi:10.1038/1765.

Watson, G. W., and Andley, U. P. (2010). Activation of the unfolded protein response by a cataract-associated αA-crystallin mutation. *Biochem. Biophys. Res. Commun.* 401, 192–6. doi:10.1016/j.bbrc.2010.09.023.

Xi, J., Bai, F., Gross, J., Townsend, R. R., Menko, A. S., and Andley, U. P. (2008). Mechanism of small heat shock protein function in vivo: a knock-in mouse model demonstrates that the R49C mutation in alpha A-crystallin enhances protein insolubility and cell death. *J. Biol. Chem.* 283, 5801–14. doi:10.1074/jbc.M708704200.

Xia, C., Liu, H., Chang, B., Cheng, C., Cheung, D., Wang, M., et al. (2006). Arginine 54 and Tyrosine 118 residues of {alpha}A-crystallin are crucial for lens formation and transparency. *Invest. Ophthalmol. Vis. Sci.* 47, 3004–10. doi:10.1167/iovs.06-0178.

Xia, X.-Y., Wu, Q.-Y., An, L.-M., Li, W.-W., Li, N., Li, T.-F., et al. (2014). A novel P20R mutation in the alpha-B crystallin gene causes autosomal dominant congenital posterior polar cataracts in a Chinese family. *BMC Ophthalmol.* 14, 108. doi:10.1186/1471-2415-14-108.

Yalcintepe, S., Gurkan, H., Gungor Dogan, I., Demir, S., Ozemri Sag, S., Manav Kabayegit, Z., et al. (2021). The Importance of Multiple Gene Analysis for Diagnosis and Differential Diagnosis in Charcot Marie Tooth Disease. *Turk. Neurosurg.* doi:10.5137/1019-5149.JTN.33661-21.3.

Yang, X. D., Cen, Z. D., Cheng, H. P., Shi, K., Bai, J., Xie, F., et al. (2017). L-3-n-Butylphthalide Protects HSPB8 K141N Mutation-Induced Oxidative Stress by Modulating the Mitochondrial Apoptotic and Nrf2 Pathways. *Front Neurosci* 11, 402. doi:10.3389/fnins.2017.00402.

Yang, Z., Su, D., Li, Q., Ma, Z., Yang, F., Zhu, S., et al. (2013). A R54L mutation of CRYAA associated with autosomal dominant nuclear cataracts in a Chinese family. *Curr. Eye Res.* 38, 1221–8. doi:10.3109/02713683.2013.811260.

Ylikallio, E., Johari, M., Konovalova, S., Moilanen, J. S., Kiuru-Enari, S., Auranen, M., et al. (2014). Targeted next-generation sequencing reveals further genetic heterogeneity in axonal Charcot-Marie-Tooth neuropathy and a mutation in HSPB1. *Eur. J. Hum. Genet.* 22, 522–7. doi:10.1038/ejhg.2013.190.

Ylikallio, E., Konovalova, S., Dhungana, Y., Hilander, T., Junna, N., Partanen, J. V, et al. (2015). Truncated HSPB1 causes axonal neuropathy and impairs tolerance to unfolded protein stress. *BBA Clin.* 3, 233–42. doi:10.1016/j.bbacli.2015.03.002.

Yu, L., Liang, Q., Zhang, W., Liao, M., Wen, M., Zhan, B., et al. (2019). HSP22 suppresses diabetes-induced endothelial injury by inhibiting mitochondrial reactive oxygen species formation. *Redox Biol* 21, 101095. doi:10.1016/j.redox.2018.101095.

Yu, Y., Xu, J., Qiao, Y., Li, J., and Yao, K. (2021). A new heterozygous mutation in the stop codon of CRYAB (p.X176Y) is liable for congenital posterior pole cataract in a Chinese family. *Ophthalmic Genet.* 42, 139–143. doi:10.1080/13816810.2020.1855665.

Zhang, H., Rajasekaran, N. S., Orosz, A., Xiao, X., Rechsteiner, M., and Benjamin, I. J. (2010). Selective degradation of aggregate-prone CryAB mutants by HSPB1 is mediated by ubiquitin-proteasome pathways. *J. Mol. Cell. Cardiol.* 49, 918–30. doi:10.1016/j.yjmcc.2010.09.004.

Zhang, L.-Y., Yam, G. H.-F., Tam, P. O.-S., Lai, R. Y.-K., Lam, D. S.-C., Pang, C.-P., et al. (2009). An alphaA-crystallin gene mutation, Arg12Cys, causing inherited cataract-microcornea exhibits an altered heat-shock response. *Mol. Vis.* 15, 1127–38. Available at: http://www.ncbi.nlm.nih.gov/pubmed/19503744.

Zhang, L., Zhang, Y., Liu, P., Cao, W., Tang, X., and Su, S. (2011). Congenital anterior polar cataract associated with a missense mutation in the human alpha crystallin gene CRYAA. *Mol. Vis.* 17, 2693–7. Available at: http://www.ncbi.nlm.nih.gov/pubmed/22065922.
